# Design of Zn‐Binding Peptide(s) from Protein Fragments

**DOI:** 10.1002/cbic.202401014

**Published:** 2025-02-26

**Authors:** Ján Michael Kormaník, Daniel Herman, Erik Andris, Martin Culka, Ondrej Gutten, Milan Kožíšek, Lucie Bednárová, Pavel Srb, Václav Veverka, Lubomír Rulíšek

**Affiliations:** ^1^ Institute of Organic Chemistry and Biochemistryof the Czech Academy of Sciences Flemingovo náměstí 2 166 10 Prague 6 Czech Republic

**Keywords:** Zinc(II), Metal-binding peptide, Computer design, Isothermal calorimetry, NMR, QM modeling

## Abstract

We designed a minimalistic zinc(II)‐binding peptide featuring the Cys_2_His_2_ zinc‐finger motif. To this aim, several tens of thousands of (His/Cys)‐X_*n*_‐(His/Cys) protein fragments (*n*=2–20) were first extracted from the 3D protein structures deposited in Protein Data Bank (PDB). Based on geometrical constraints positioning two Cys (C) and two His (H) side chains at the vertices of a tetrahedron, approximately 22 000 sequences of the (H/C)‐X_*i*_‐(H/C)‐X_*j*_‐(H/C)‐X_*k*_‐(H/C) type, satisfying *N*_metal–binding H_=*N*_metal‐binding C_=2, were processed. Several other criteria, such as the secondary structure content and predicted fold stability, were then used to select the best candidates. To prove the viability of the computational design experimentally, three peptides were synthesized and subjected to isothermal calorimetry (ITC) measurements to determine the binding constants with Zn^2+^, including the entropy and enthalpy terms. For the strongest Zn^2+^ ions binding peptide, **P1**, the dissociation constant was shown to be in the nanomolar range (*K_D_* =~220 nM; corresponding to Δ*G*_bind_=−9.1 kcal mol^−1^). In addition, ITC showed that the [**P1** : Zn^2+^] complex forms in 1 : 1 stoichiometry and two protons are released upon binding, which suggests that the zinc coordination involves both cysteines. NMR experiments also indicated that the structure of the [**P1** : Zn^2+^] complex might be quite similar to the computationally predicted one. In summary, our proof‐of‐principle study highlights the usefulness of our computational protocol for designing novel metal‐binding peptides.

## Graphical abstract

We have designed a short zinc‐binding peptide by taking fragments from proteins with known structure. We have measured the binding affinity with isothermal calorimetry and the structure of the peptide using CD and NMR spectroscopies. These results were computationally supported by molecular dynamics and density functional theory calculations.

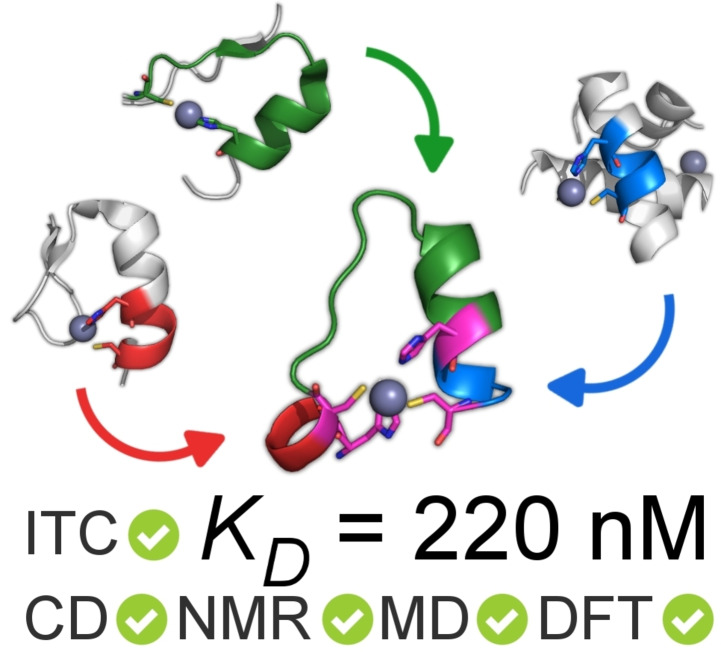

## 1 Introduction

Metalloproteins represent around 35 % of known protein structures.[^1^, ^2^, ^3^] Metal ions presented therein fulfill a variety of functions. Not only do they play a structural role, ensuring proper folding and shape of metalloproteins, but often they are also responsible for their catalytic activity.^[4]^ Metalloproteins are implicated in redox processes in living cells,^[5]^ spin‐forbidden reactions,^[6]^ gas transfer,^[7]^ heavy metal detoxification,^[8]^ signal transduction,^[4]^ as well as metal ion storage.^[7]^ Thus, it comes as no surprise that metalloproteins not only inspired development of many small‐molecule catalysts,[^9^, ^10^] but also served as starting points for enzyme redesign or *de novo* design.[^11^, ^12^, ^13^, ^14^]

Zinc fingers (ZFs) are an example of small metalloprotein domains composed of 20–30 amino acids (AAs), often appearing as a repeating unit in larger proteins. ZFs bind a zinc ion that enhances their structural stability.^[15]^ The binding site is mostly tetrahedral, harboring {C_2_H_2_} or {C_4_} coordination motives, although similar domains, like {C_3_H} or domains binding multiple zinc ions including {C_6_H_2_} or {C_7_H}, are also recognized as ZFs.[^16^, ^17^] ZF‐containing proteins represent up to ~5 % of protein‐coding genes in the human genome[^17^, ^18^] and are involved in various interactions with DNA, RNA, and other proteins, giving rise to their involvement in biological processes such as transcriptional regulation, protein degradation, DNA repair, or cell differentiation.[^16^, ^17^, ^19^] ZFs are thus attractive targets for bioengineering efforts.^[17]^ The relatively small lengths of ZFs have also made them an interesting target for various studies, both structural and functional, including protein design efforts.[^4^, ^17^] Designs based upon ZFs include short domains that switch folds depending on zinc concentration,[^20^, ^21^] small hydrolytic metallopeptides,[^17^, ^22^] or ZFs binding various metal ions other than zinc.^[17]^ Such design efforts, often supported computationally, prove the viability of ZFs for various purposes.

Computational *de novo* design of metalloproteins requires full understanding of the interactions between the metal ion and the peptide/protein framework. This understanding comprises two parts: (i) designing the peptide scaffold that may easily adopt the target metal‐coordination geometry, and (ii) predicting the metal‐binding affinity or selectivity (i. e., relative affinity) of general {X_*n*_} site for a selected metal. As for the latter, the affinities can be either qualitatively derived from the experimental stability constants of similar systems or computed from first principles. To this aim, our group formulated a robust computational protocol to theoretically predict metal‐ion stability constants in peptide/protein sites.[^23^, ^24^] Recently, we used the same protocol, based on the DFT‐D3//COSMO‐RS calculations, to quantitatively predict binding constants of Pb(II), Ca(II), and Zn(II) in various designed lead‐binding peptides.[^25^, ^26^, ^27^] Concerning the scaffold design, it must be mentioned that in the last couple of years, major breakthroughs revolutionized protein structure predictions. They may also, at least partially, aid in designing new peptides. Among these breakthroughs, we may highlight DeepMind's AlphaFold 2/3,[^28^, ^29^] which has come close to the solution of the enigmatic protein structure prediction problem; UNRES Web Server,^[30]^ which was designed to do conformational sampling of intrinsically disordered proteins; as well as Rosetta software suite developed by Baker Lab, including RFdiffusion for generating protein backbones^[31]^ and ProteinMPNN for sequence design.^[32]^ Modelling the metal ion as a tetrahedral ligand has also been shown to improve the scaffold structure prediction, although this requires prior knowledge of binding residues.^[33]^

Nowadays, various conceptually different approaches to the design of metallopeptides exist, including protein redesigns and *de novo* designs, for binding either metal ions or metal‐containing coenzymes.[^13^, ^34^, ^35^] Among recent advances in this field, we can find coiled coils,^[36]^ metalloproteins with non‐native backbones built from α‐helices,^[37]^ modified β‐hairpins[^38^, ^39^] and WW domains,[^40^, ^41^] oligomeric protein assemblies with pre‐designed metal‐binding sites[^31^, ^42^] or even short self‐assembling peptides with catalytic activity.^[43]^ All these designs exploit the knowledge of preferred binding sites of various metals. It is also possible to use these approaches to design proteins binding uncommon metal ions, such as lanthanoids,^[44]^ further expanding the usual scope of metalloproteins and opening new paths for their potential applications. However, metal ions offer much more than just binding for structural stability. Catalytically active metalloproteins have also been designed. Recent reports include a di‐nickel hydrogenase designed from short fragments^[45]^ or di‐copper center with catechol oxidase activity.^[46]^ Catalytic activity of all designed metalloenzymes still lags behind natural enzymes. Nevertheless, these results are inspiring for future research. All this underscores the future potential applications of designed (catalytic) metalloproteins or metallopeptides.

In this work, we aim to explore the potential of computational methods in the *de novo* design of a metal‐binding peptide with zinc‐finger motif {C_2_H_2_}. The strategy was conceptually straightforward: (1) we extracted (H/C)‐X_*n*_‐(H/C) fragments from protein structures in the Protein Data Bank (PDB) and (2) we merged them geometrically into a single chain featuring two C and two H metal‐binding residues in ideal positions. We have attempted a similar, albeit much simpler approach previously,^[47]^ which resulted in 20‐peptide denoted HHTC (to acknowledge the four plausible metal‐binding residues), with ~400 nM copper(II)‐ and ~10 μM zinc(II)‐binding affinity.^[47]^ Herein, more advanced, versatile, and iterative protocol has been developed that led to the selection of three peptides for experimental verification of our computational model. The peptides (**P1**, **P2**, and **P3**) were synthesized and the thermodynamics of the zinc(II) binding was assessed by isothermal calorimetry (ITC). The structure of the [metal : peptide] complex was elucidated by nuclear magnetic resonance spectroscopy (NMR) accompanied by electronic circular dichroism (ECD) spectroscopy. The best of the *de novo* designed peptides, peptide **P1**, showed ~220 nM zinc(II)‐binding affinity, which is 50‐fold improvement over our previous work. This dissociation constant is still at least a few orders of magnitude higher in comparison to their natural ZF counterparts which exhibit affinities near or below low nanomolar range.^[15]^ Still, these results demonstrate the viability of our original design approach and provide guidelines for future improvement.

## 2 Results

### 2.1 PDB Fragment Libraries: Design Principle

The aim of this study is to determine whether it is possible to find new peptide sequences/folds, in addition to the well‐established ones (nicely summarized for example in the Refs.[^48^, ^49^, ^50^]), that would fold and bind a metal ion in a proposed binding site and geometry, by merging several fragments from proteins with known structure. The metal center is then expected to provide additional stabilization of the protein/peptide structure and may promote its folding into the metal‐binding geometry. We selected a tetrahedral binding site comprising the {C_2_H_2_} zinc‐finger motif, thus featuring the {C_2_H_2_} side chains at the vertices of the tetrahedron, that is assumed to accommodate most of the divalent metal ions once the peptide chain would fold into the proper geometry.

For this purpose, we searched for the **B**_*i*_‐X_*n*_‐**B**_*j*_ sequences (where X_*n*_ denotes a peptide sequence of the length *n*; **B**_*i/j*_ are metal‐binding residues, in our case always **B**_*i/j*_=H or C; and we requested 1<*n*<21) in any protein in the bc‐100 database, which contained 67,359 PDB structures/files. In addition, we requested the boundary condition that the ‘distance’ between the first **B**_*i*_ (**B**_*i*_=H/C) residue and the last (**B**_*j*_) residue in the **B**_*i*_‐X_*n*_‐**B**_*j*_ sequence/fragment is not greater than 6.5 Å. This ‘residue distance’ is defined by donor atoms of the binding terminal residues (sulfur for cysteine and imidazole nitrogens for histidine).

**Figure 1:**
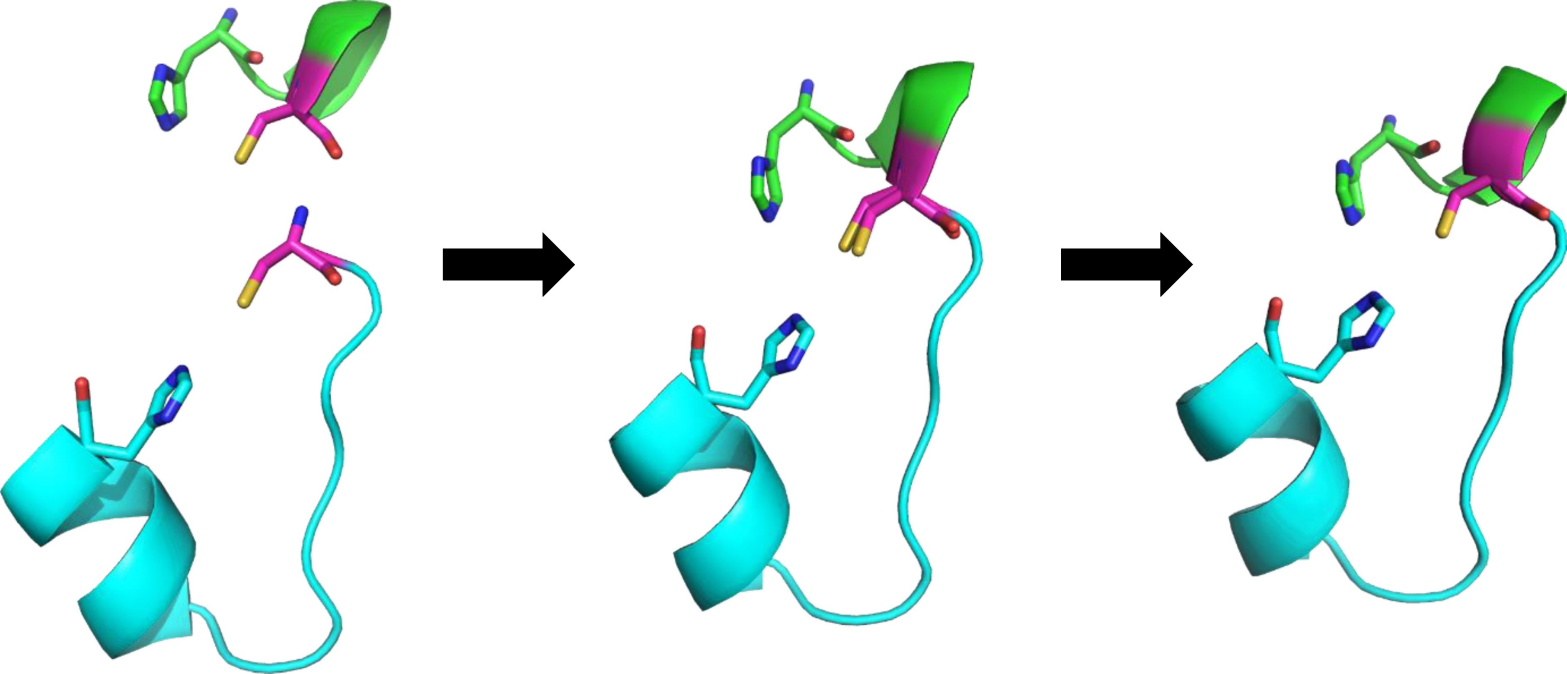
Merging two fragments into a larger peptide. When we find two compatible fragments (left), the terminal AA of the first fragment is overlaid onto the first AA of the second fragment (center). We only consider the 10 best alignments according to the clustering by the RMSD. These fragments are then merged into a new peptide and only the C‐terminal AA from the first fragment is kept in the new peptide (right). Similar process is then repeated to obtain the final design. The two fragments are shown in cartoon in lime and cyan, respectively, the shared cysteine is shown in magenta. All terminal binding AAs are shown as sticks, hydrogens are omitted for clarity.

Having this **B**_*i*_‐X_*n*_‐**B**_*j*_ data set, the next step aimed at merging the fragments into a single **B**_1_‐X_*k*_‐**B**_2_‐X_*l*_‐**B**_3_‐X_*m*_‐**B**_4_ polypeptide sequence (see Figure 1).

To reduce the enormous number of possibilities, which would be computationally intractable, we only considered fragments with similar structural arrangement of the overlapping AAs (**B**_*i*_). When merging two fragments, **B**_1_‐X_*k*_‐**B**_2_ and **B’**_2_‐X_*l*_‐**B**_3_, we request that the last AA from the first fragment (**B**_2_) must be identical to **B’**_2_ (i. e., both are either C or H), and also have similar conformations of the main chain. Specifically, for any **B**_1_‐X_*k*_‐**B**_2_ fragment, all possible **B’**_2_‐X_*l*_‐**B**_3_ fragments are aligned, employing primarily N, Cα, and C atoms of **B**_2_ and **B’**_2_. All **B’**_2_‐X_*l*_‐**B**_3_ fragments have been clustered using Locality–Sensitive Hashing forest (LSH forest) in order to find the 10 closest alignments (ordered by RMSD of the alignment) of this overlapping AA (**B**_2_=**B’**_2_). For RMSD calculation during clustering, the same three backbone atoms were considered, as well as the binding atoms (sulfur for cysteine and imidazole nitrogens for histidine). We then took the top 10 alignments, for which the fragments were joined and the terminal AA from the first fragment (**B**_2_) was kept, resulting in the **B**_1_‐X_*k*_‐**B**_2_‐X_*l*_‐**B**_3_ peptide with potentially three metal‐binding residues (see Figure 1).

In the last step of this process, we considered **B**_1_‐X_*k*_‐**B**_2_‐X_*l*_‐**B**_3_ and **B’**_2_‐X_*l*_‐**B’**_3_‐X_*m*_‐**B**_4_ fragments, which share the fragment **B**_2_‐X_*l*_‐**B**_3_=**B’**_2_‐X_*l*_‐**B’**_3_ (sequence‐wise) to obtain a model for the final **B**_1_‐X_*k*_‐**B**_2_‐X_*l*_‐**B**_3_‐X_*m*_‐**B**_4_ peptide. We then checked the shape of the potential {**B**_1_, **B**_2_, **B**_3_, **B**_4_} binding site. The distance between any pair of donor (metal‐binding) atoms was requested to be between 1.5 and 5.5 Å and it can be reminded that we limited our design to {C_2_H_2_} sites. We have obtained 21,926 peptides for further processing. Once again, it must be re‐emphasized that all **B**_*i*_‐X_*n*_‐**B**_*j*_ fragments used in our design are taken from *any* protein with known structure.

### 2.2 Refining the Designed Metallopeptides

To select candidates for experimental measurements from the almost 22,000 (H_1_/C_1_)‐X_*k*_‐(H_2_/C_2_)‐X_*l*_‐(H_3_/C_3_)‐X_*m*_‐(H_4_/C_4_) peptide candidates (see Section 2.1.), we used two rational approaches: (i) secondary structure content of these peptides, to ensure at least partial folding, and (ii) molecular dynamics (MD), to assess stability of the peptides.

We presumed that secondary structure in the designed peptides could help with holding the overall structure of the peptide, improving the chances of a stable metal binding site. We thus assessed the secondary structure of each designed peptide using the STRIDE program^[51]^; note that the fragments are taken from proteins in PDB with known three‐dimensional structure. We have decided to only consider peptides that start and end with a helix (or those that have a short coil, up to four AAs, before the first and after the last helix). This condition has been rather arbitrary, based on the fact that a handful of stable helix‐loop‐helix folds of oligopeptides were reported in the literature[^52^, ^53^] and this motif is common in transcriptional factors, for example.^[54]^ In addition, we also required the sequences to be unique (thanks to the inherent PDB redundancy, there have been many identical sequences among the original set of 22,000 peptides). Applying the two criteria, we reduced the number of peptides to 101.

We further assessed all 101 of these peptides using MD to test the stability of their fold both with and without zinc ion. For details of the MD runs, see Methods. During the MD runs, we mapped the change in RMSD of the peptide with respect to the starting (originally designed) structure in the *apo* peptides. In their complexes with zinc(II) ion, we also monitored the M−X distances throughout the MD trajectory, admitting that force field calculations for Zn^2+^ sites are more on the approximate side.^[55]^ The runs were then visually inspected and the peptides which unfolded (in their metal‐bound form) or peptides where the zinc ion escaped from the binding site were not considered further.

**Figure 2:**
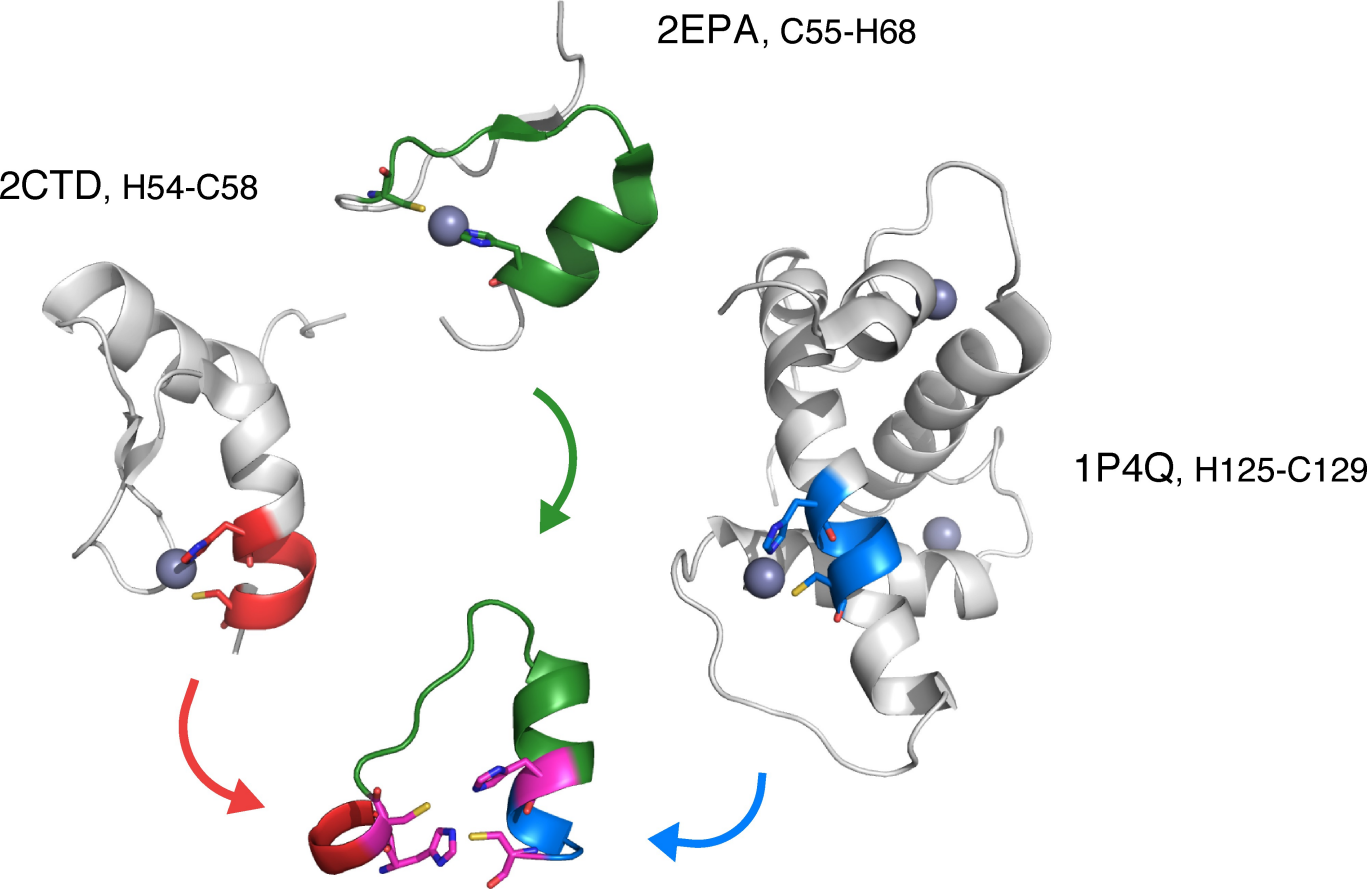
Source proteins for peptide **P1**. Each fragment is colored according to the source PDB with the proposed binding residues shared by two fragments in magenta. Only parts of the proteins from PDB are shown. Proteins are shown by cartoon with binding residues as sticks, hydrogens are omitted for clarity.

Based on the MD runs and the overall fold stability, we have selected three peptides, **P1**, **P2**, and **P3**. These peptides are composed of the following fragments: **P1** is composed of fragments from PDB entries 2CTD (H54‐C58), 2EPA (C55‐H68), and 1P4Q (H125‐C129); **P2** is composed of fragments from PDB entries 2CT2 (H39‐C59), 2EOD (C195‐H210), and 2EOD (H210‐C214); and peptide **P3** is composed of fragments from PDB entries 2YSJ (H40‐C60), 1WJV (C13‐H28), and 1WJV (H28‐C32). The ‘PDB origin’ of **P1** is shown in Figure 2.

For each of the peptides, we have also prepared alternatives (**P1C**, **P2C**, and **P3C**, respectively). These peptides were used as a control – the designed peptides (**P1**–**P3**) should be better zinc binders than the corresponding control peptides. For **P1**, we obtained the new sequence by permuting the original sequence. Specifically, we kept the proposed metal‐binding residues in place and for each fragment connecting the binding residues, we randomly permuted the sequence of AAs within the given fragment. For peptides **P2** and **P3**, we substituted certain cysteines by serines which also included one of the metal‐binding cysteines. Admittedly, this might not have been the best strategy as it presumably interfered with the metal‐binding affinity of the **P2C** and **P3C** peptides. Viewed retrospectively, C24 in both **P2** and **P3** should have been better candidates. The sequences of both designed and control peptides are shown in Table 1.

**Table 1:** Sequences of the designed and control peptides, (presumed) binding AAs are shown in red and bold.

Peptide	Sequence
**P1** **P1C** **P2** **P2C** **P3** **P3C**	Ac‐**H**MEN**C**ERRFARSDELSR**H**AHK**C**‐NH_2_ Ac‐**H**NME**C**LFARSSEDRRER**H**HKA**C**‐NH_2_ Ac‐**H**TI**C**RQ**C**LEKLLASSINGVR**C**TY**C**TKEFVFDTIQS**H**QYQ**C**‐NH_2_ Ac‐**H**TI**S**RQ**S**LEKLLASSINGVR**S**TY**C**TKEFVFDTIQS**H**QYQ**C**‐NH_2_ Ac‐**H**NF**C**LK**C**ITQIGETS**C**GFFK**C**NA**C**GESVKKIQVEK**H**VSN**C**‐NH_2_ Ac‐**H**NF**S**LK**S**ITQIGETS**S**GFFK**S**NA**C**GESVKKIQVEK**H**VSN**C**‐NH_2_

### 2.3 Thermodynamics of Metal Binding

We used isothermal titration calorimetry (ITC) to monitor the binding of Zn^2+^ ion to the designed and control peptides. However, during experiments, peptides **P2**, **P2C** and **P3** demonstrated non‐ideal behavior and were therefore excluded from the study. Peptides **P2** and **P2C** were insoluble in the buffer at concentrations required for calorimetric measurements, while peptide **P3** exhibited ion‐binding stoichiometry greater than one, as seen in Figure S1 in the SI. All experimental thermodynamic parameters for the peptides **P1** and **P1C** are summarized in Table 2, the thermodynamic parameters of peptide **P3C** are in Table S1 in the SI.

**Table 2:** Thermodynamic parameters of Zn^2+^ ion binding to peptides **P1** and **P1C**.

Peptide	Δ*G*_bind_ [kcal mol^−1^]	Δ*H*_bind_ [kcal mol^−1^]	‐*Τ*⋅Δ*S*_bind_ [kcal mol^−1^]	*K_A_* [M^−1^]	*K_D_* [nM]	Stoichiometry	*n*_H+_
**P1**	−9.1±0.2	−2.9±0.6	−6.2±0.8	(4.5±1.5) ⋅ 10^6^	220±90	0.99±0.05	−1.8±0.1
**P1C**	−8.6±0.1	−3.7±0.3	−4.9±0.4	(1.9±0.3) ⋅ 10^6^	520±70	0.96±0.09	−1.5±0.1

The titrations of **P1**, **P1C**, and **P3C** performed in buffers with different enthalpies of ionization Δ*H*_ion_ (ACES, HEPES, PIPES, and sodium cacodylate) yielded different experimentally observed binding enthalpies, indicating that under the experimental conditions, there is a net proton transfer coupled to Zn^2+^ ion binding to peptides (Figures S2–S4 in the SI).[^56^, ^57^] Stoichiometries of the complexes were close to 1 (one Zn^2+^ ion to one peptide molecule).

**Figure 3:**
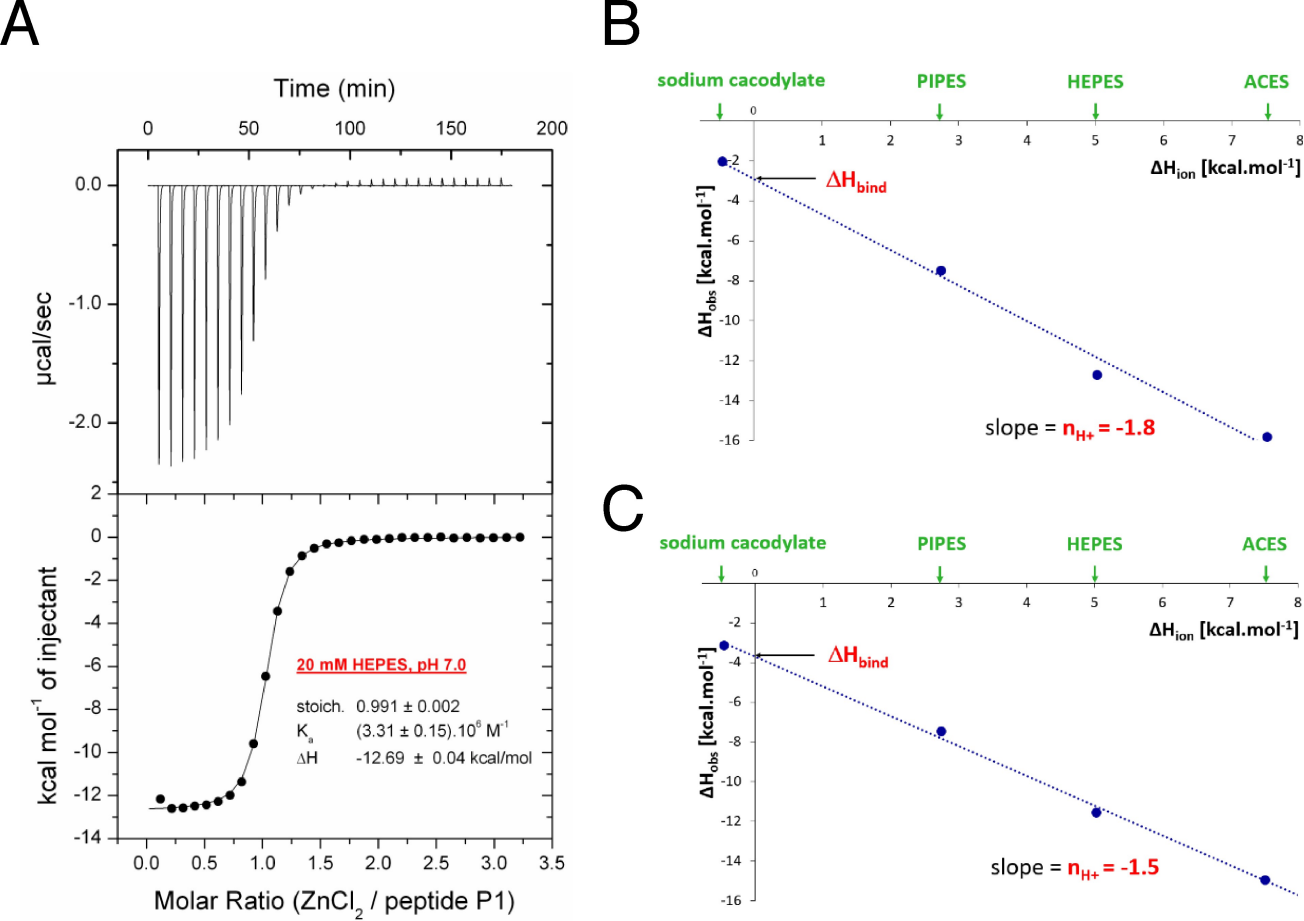
(**A**) An example of isothermal titration of ZnCl_2_ to peptide **P1** performed in 20 mM HEPES, pH 7.0 at 298.15 K. Experimentally observed binding enthalpies (Δ*H*_obs_) from titration of ZnCl_2_ to peptide **P1** (**B**) and **P1C** (**C**) in four different buffers, plotted against enthalpy of ionization of each buffer (Δ*H*_ion_).

The dissociation constant (*K_D_*) corresponding to the binding of Zn^2+^ ion to peptide **P1** is 220 nM, which corresponds to Gibbs energy of binding Δ*G*_bind_=−9.1 kcal mol^−1^ (with buffer corrected enthalpic term Δ*H*_bind_=−2.9 kcal mol^−1^ and entropic term −*T* ⋅ Δ*S*_bind_=−6.2 kcal mol^−1^, at *T*=298.15 K). When zinc binds to **P1**, 1.8 protons are released, which we interpret as both cysteines being involved in binding Zn^2+^ ion (see Figure 3).

Similarly, Zn^2+^ ion binds to **P1C** with the dissociation constant of 520 nM, which corresponds to Δ*G*_bind_=−8.6 kcal mol^−1^, Δ*H*_bind_=−3.7 kcal mol^−1^, and −*T*⋅Δ*S*_bind_=−4.9 kcal mol^−1^, at *T*=298.15 K. The complex formation was accompanied by the release of 1.5 protons.

### 2.4 Structural Characterization: Electronic Circular Dichroism

Having established that **P1** and **P1C** are good binders of Zn^2+^, we focused on structural characterization of the peptides with and without Zn^2+^. For this purpose, we used electronic circular dichroism (ECD) spectroscopy as a tool for fast determination of secondary (far‐UV spectral region, 190–250 nm) and tertiary (near‐UV spectral region, 250–300 nm) structure of peptides and proteins.

**Figure 4:**
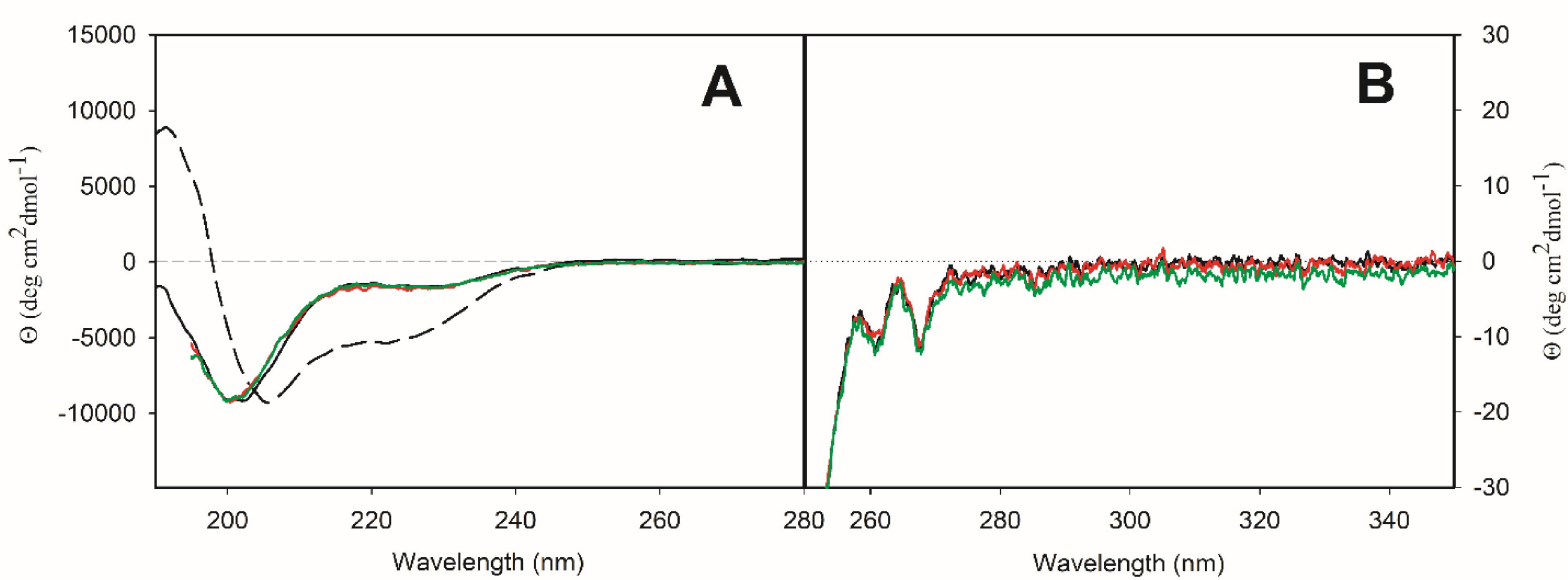
ECD spectra of peptide **P1** in buffer solution (solid black curve) and 25 % (v/v) TFE (dashed black curve) and of [**P1** : Zn^2+^] complex in water in stoichiometry 1 : 1 (red curve) and 1 : 2 (green curve) in far‐UV (**A**) and near‐UV (**B**) spectral region.

ECD spectra in both spectral regions of peptide **P1** without and with Zn^2+^, including the excess of zinc ions, are rather similar (Figure 4). The negative spectral band at ~201 nm in CD spectra in far‐UV spectral region of **P1** without and with Zn^2+^ (Figure 4A) reflects mostly disordered structure (~33 %, see Table S2 in the SI). The negative shoulder at ~228 nm could reflect the presence of α‐helical structure or the presence of β‐sheet structure. The latter was confirmed by numerical spectral analysis (Table S2 in the SI). In the near‐UV spectral region, the CD spectra of **P1** without and with Zn^2+^ show negative spectral bands with the same intensity at ~261 nm and ~268 nm, which are characteristic spectral bands for phenylalanine. The interaction with Zn^2+^ thus does not cause any significant changes in the secondary structure content of **P1**. The ability of **P1** to adopt α‐helical conformation was tested by adding 2,2,2‐trifluoroethanol (TFE), known as an α‐helix‐inducing solvent. In the presence of 25 % (v/v) TFE, we observed an increase of α‐helical content at the expense of β‐sheet content (Table S2 in the SI). The partition of other secondary structures (disordered and β‐turn) did not change significantly.

**Figure 5:**
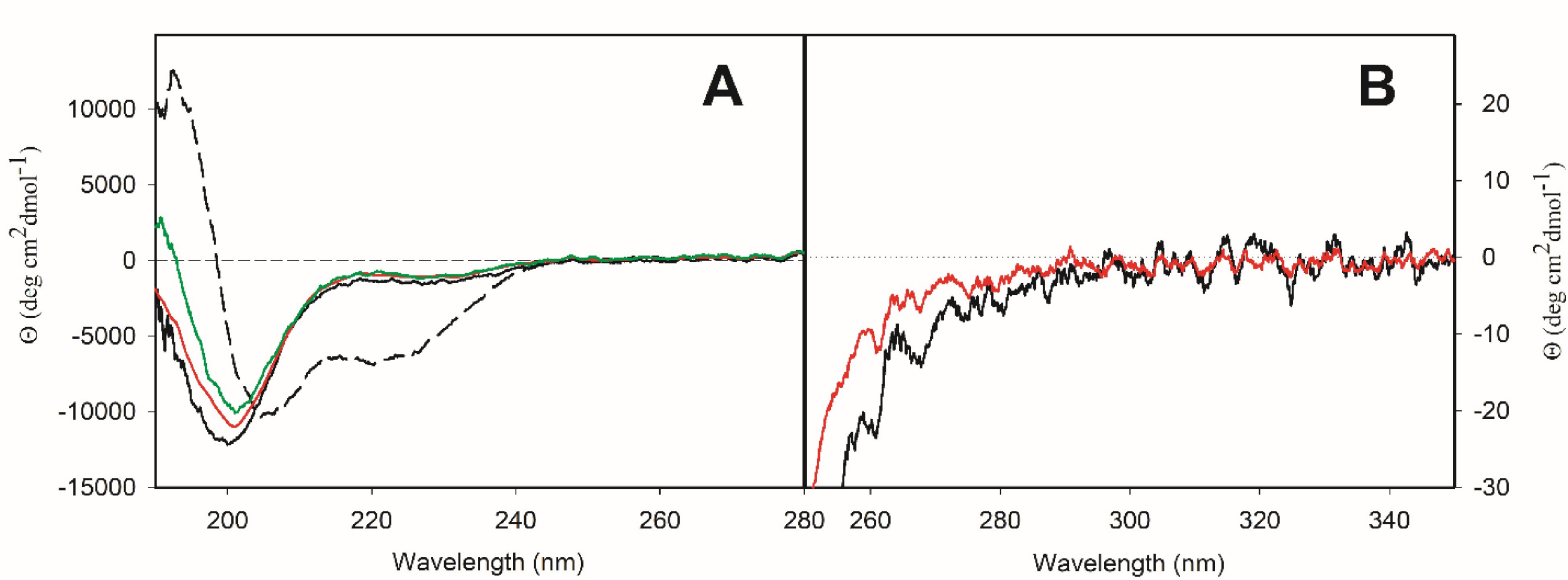
ECD spectra of peptide **P1C** in buffer solution (solid black curve) and 25 % (v/v) TFE (dashed black curve) and of [**P1C** : Zn^2+^] complex in water in stoichiometry 1 : 1 (red curve) and 1 : 2 (green curve) in far‐UV (**A**) and near‐UV (**B**) spectral region.

The structure of control peptide **P1C** without and with Zn^2+^ was also characterized by ECD (Figure 5). The general pattern of secondary structure of **P1C** without and with Zn^2+^ was comparable to **P1**. The higher intensity of negative spectral band at ~201 nm hints at a higher content of disordered secondary structure, which was confirmed by numerical analysis of experimental CD spectra (Table S2 in the SI). In the near‐UV spectral region, the CD spectra of **P1C** without and with Zn^2+^ show negative spectral bands at ~261 nm and ~268 nm, but these bands are less pronounced compared to **P1** and, in presence of Zn^2+^, have an even lower intensity. Thus, though the secondary structure of **P1** and **P1C** seem very similar, the peptide **P1C** adopts a less defined and more flexible structure compared to **P1**. In the presence of 25 % (v/v) TFE, **P1C** adopted similar partition of α‐helical structure as **P1**, but according to numerical analysis, the content of disordered structure was lower at the expense of β‐turn structure when compared to **P1**.

### 2.5 Three‐Dimensional Structure: NMR

**Figure 6:**
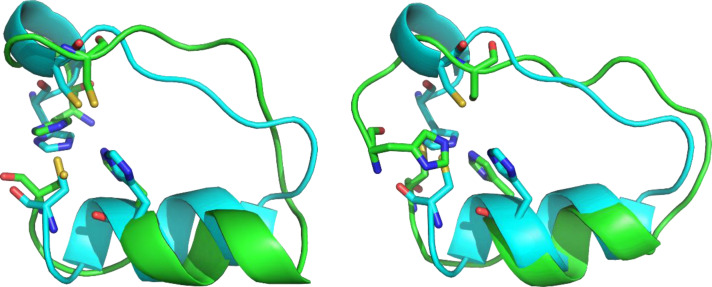
Comparison of the NMR models (lime) with the designed peptide **P1** (cyan; shown without zinc). Using the restraints from NMR and ITC, 20 models of the peptide were generated. Here we show the best model (left, all‐atom RMSD=2.9 Å) and the median of the 20 models (right, all‐atom RMSD=4.1 Å) according to the all‐atom RMSD. Peptides are shown by cartoon with binding residues shown as sticks, hydrogens are omitted for clarity.

We utilized NMR spectroscopy to elucidate structural features of **P1** and the [**P1** : Zn^2+^] complex and their comparison with the predicted design. The NMR sample was prepared using synthesized **P1** and a series of spectra was acquired as described in Methods. We used the obtained chemical shift assignments of all AAs of **P1** and [**P1** : Zn^2+^] for tracing secondary structure elements. In the bound state, [**P1** : Zn^2+^], we observed a significant propensity for α‐helical conformation for residues D13–A19 of the peptide, whereas the rest of the residues are defined as dynamic. This agrees quite well with the designed peptide (and MD simulations, see below), where the longest α‐helix comprises residues S12‐H20. It can be mentioned that S12–H20 originates from the structure with PDB code 2EPA. The analysis of NOESY cross peaks provided 145 distance restraints in total (Table S3) with 8 important long‐range restraints connecting F9 aromatic protons to E14 and L15 side chain protons (Table S4). Stabilization of this part of the molecule leads to the formation of the hydrophobic core of our peptide. Unfortunately, we did not get any NMR evidence for contacts between the zinc‐binding residues (H1, C5, H18, and C22). As the ITC measurements strongly suggest that at least two out of the four metal‐binding residues of **P1** are interacting with Zn^2+^ ion, the lack of peaks for these contacts likely corresponds to a significant conformational flexibility of the chain termini. We note in passing that we also performed exploratory measurements of oxidized form of **P1**, with covalently linked C5 and C22, and likewise did not observe any such cross‐peaks (data not shown). Also, a qualitative comparison of 2D NOESY spectra of [**P1** : Zn^2+^] with **P1** (see Figure S5 in the SI) shows appearance of many cross‐peaks upon Zn^2+^ binding, which agrees with fold stabilization. Using these restraints, we have created several NMR models of our peptide. Comparison of selected NMR models to the original design can be seen in Figure 6. According to the NMR models, however, the binding site can also change between two geometrical isomers (Figure S6 in the SI). Both isomers can be present in solution and potentially interconvert between one another, which would partially explain why we did not observe any contacts between the zinc–binding residues in the NMR spectra. In the unbound state of **P1**, α‐helical conformation is similarly observed for residues D13‐A19. One of the NMR models of the unbound **P1** is shown in Figure S7 in the SI. This also suggests that the central part of the peptide is preorganized and upon zinc binding, the peptide folds in half, allowing the termini to create a binding site.

Therefore, NMR data confirm that the central part of **P1** is in line with our predicted design. Both termini, involving two of the Zn^2+^‐binding residues, are too flexible in the *apo* form to tell anything about the structure, except that Zn^2+^ ion binding significantly affects (and likely stabilizes) the structure. Unfortunately, for **P1C**, we were unable to solve the structure using NMR. For free **P1C**, the 1D spectrum shows more peaks than would be expected, suggesting high flexibility of the peptide or aggregation during measurement (Figure S8 in the SI). Upon addition of zinc ions and formation of the [**P1C** : Zn^2+^] complex, some peaks disappear, which suggests stabilization of the conformational flexibility or rearrangement of the formed aggregates or their dissolution and partial unfolding. Even though the binding stoichiometry is known from ITC measurements (**P1C** binds zinc ions in 1 : 1 stoichiometry), it is likely there are multiple binding conformations or that the zinc acts as a bridging ligand between two or more peptides, preventing us from solving the structure.

### 2.6 Molecular Dynamics Simulations

Peptide **P1** was subjected to 450 ns molecular dynamics (MD) simulations to probe the stability of the fold in two setups, with and without the (bound) Zn^2+^ ion (i. e., [**P1** : Zn^2+^] and **P1** forms, respectively). The two cysteine residues were deprotonated in the [**P1** : Zn^2+^] simulation. At the same time, the correct protomers of all histidine residues were selected (protonated at Nδ for both binding histidines, H1 and H18, and protonated at Nϵ for H20). No covalent bonds were defined between the Zn^2+^ ion and the protein. A similar setup was then carried out for **P1C** (see below).

**Figure 7:**
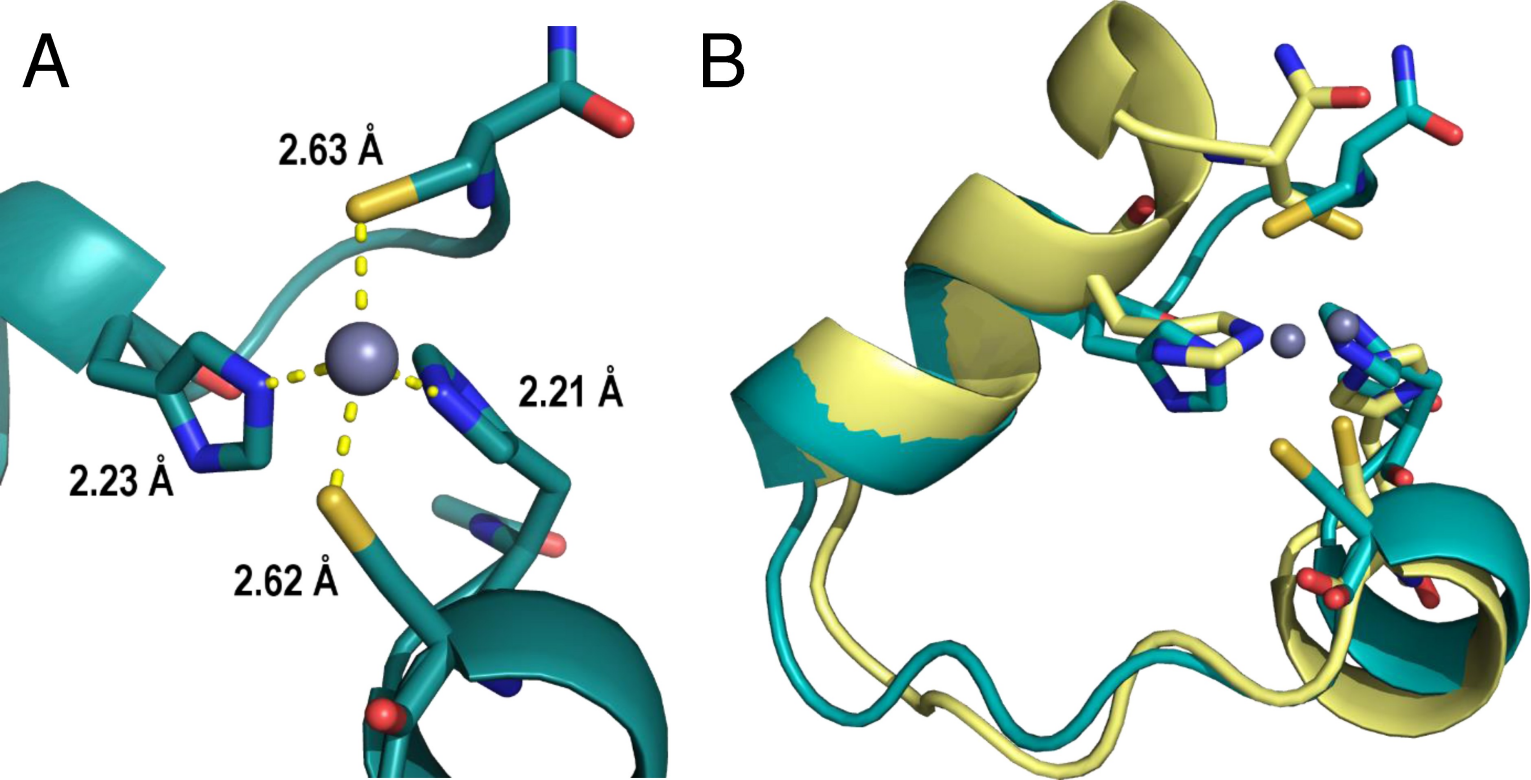
(**A**) The average distances between the metal‐binding residues and the zinc ion during MD run. (**B**) Selected frames from the MD study of [**P1** : Zn^2+^] (at 138 ns, yellow, and 434 ns, teal). While the overall shape of the binding site is very similar, the conformations of the binding residues vary, which can be seen on both the terminal cysteine residue (at the top of the figure) and relative orientation of the rings of both histidine residues. Peptide is shown by cartoon with binding residues shown as sticks and zinc ions shown as spheres, hydrogens are omitted for clarity.

The zinc ion stayed bound for the entire production trajectory (450 ns) of the [**P1** : Zn^2+^] complex and the overall fold remained essentially stable. The average distances from the zinc ion to the binding atoms throughout the run are shown in Figure 7A. The MD trajectory also clearly shows that the residues F9 and E14/L15 are close enough to show interactions within NOESY spectra (Figure S9 in the SI). While the binding site has a relatively stable shape during the MD run, the binding residues still show conformational variability. The last turn of the short C‐terminal helix occasionally unfolds, resulting in change of conformation and orientation of C22. The binding histidine residues (H1 and H18) also show great variability in the orientations of their imidazole rings relative to the rest of the peptide. An example of the difference between these conformations can be seen in Figure 7B. The distances between the side chain β‐protons of zinc‐binding residues are too long on average to be visible in NMR (Figure S10 in the SI). Both these findings would partially explain why no evidence of contacts between zinc‐binding residues is present in NMR.

In contrast, the *apo* form (peptide **P1**) starts unfolding after 100 ns of the simulation and the C‐terminal helix unfolds as the last structured part after ~200 ns. Interestingly, the peptide showed a tendency to re‐fold into a different form (Figure S11 in the SI) after 250 ns instead of remaining in the random coil state. It remained in this alternative conformation until the end of the simulation. The MD results thus show that zinc binding stabilizes the fold of **P1**.

The same study was performed for **P1C** in both free form and zinc bound [**P1C** : Zn^2+^] complex. The initial structure of **P1C** was obtained by mutating the original design of **P1** using a fixed backbone design application (*fixbb*)^[58]^ in Rosetta 3.9 with standard settings (*REF2015* scoring function).^[59]^ The backbone of the peptide was kept fixed and all non‐binding residues were mutated to the corresponding sequence. Correct protomers were selected for histidines (binding histidines, H1 and H18, were protonated at Nδ and the non‐binding histidine, H19, was protonated at Nϵ). No covalent bonds were defined between the Zn^2+^ ion and the protein.

**Figure 8:**
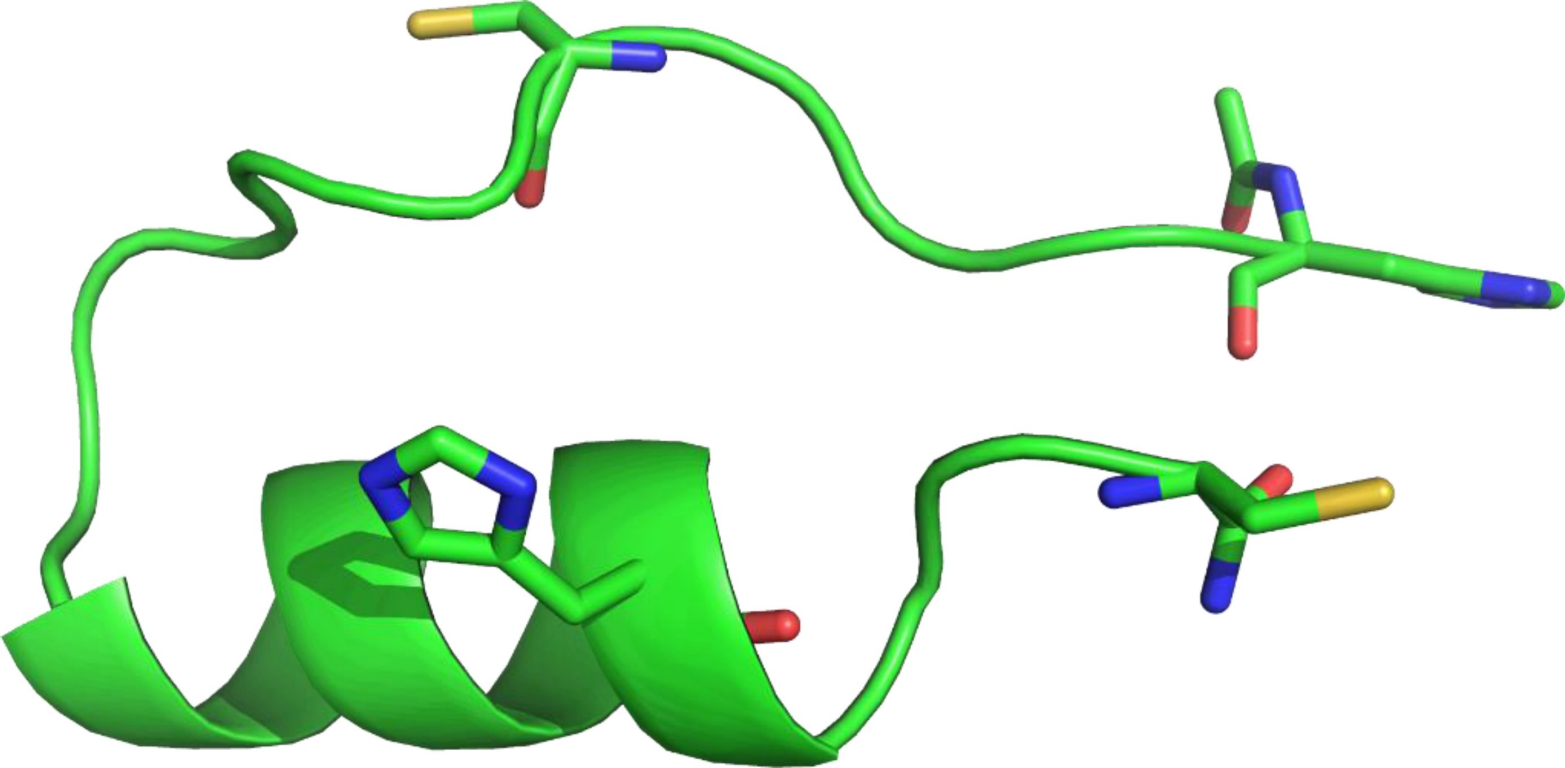
The final structure of the free **P1C** from the MD run at 450 ns. The peptide folded into a more compact conformation, where the N‐terminal section is seemingly held in place by the arginines on the C‐terminal α‐helix (residues not shown). The proposed binding residues (shown in sticks) are unable to form a tetrahedral binding site in this conformation. Peptide is shown by cartoon with presumed binding residues shown in sticks, hydrogens are omitted for clarity.

For the [**P1C** : Zn^2+^] complex, we see a behavior comparable to [**P1** : Zn^2+^], namely that the input fold remains relatively stable throughout the whole simulation (450 ns). Zinc ion stayed bound to the binding residues during the whole MD run. In the unbound form, the input structure of **P1C** first changes into linear conformation almost immediately (after 5 ns), where only the C‐terminal helix remains folded. However, after 280 ns, the peptide then folds in half and adopts a structure that is even more compact than the input structure (Figure 8), which is stable until the end of the simulation. In this compact structure, residues S11‐H19 form an α‐helix, the hydrophobic residues L6 and F7 are located on the outside and the residues E4‐A8 seem to be held in place by interactions between their carbonyl oxygens and arginines R14 and R17.

If we consider this as a stable conformation of the peptide, the hydrophobic residues facing outward might promote aggregation of the free **P1C**, which would be consistent with NMR measurements. When zinc ions are present, this would either induce slight unfolding of this compact structure so that the presumed binding residues can form a tetrahedral binding site, or the zinc ions form bridges between multiple peptides. As peaks disappear in the NMR measurements upon addition of zinc ions, unfolding is more likely, but it is possible there are multiple conformations and/or binding modes between the peptide and the zinc ions.

It should be noted, however, that the length of the analyzed MD runs for both forms of both peptides were 450 nanoseconds, which is 6 orders of magnitude shorter than the time of NMR measurements (milliseconds), thus our MD results might describe only a fraction of the behavior of the peptides.

### 2.7 QM/COSMO‐RS Calculations to Predict the [P1 : Zn^2+^] Stability Constants

Encouraged by accurate quantum chemical computations of stability constants of several divalent ions in peptides,[^25^, ^26^, ^27^] we wanted to test whether this protocol still gives quantitatively correct results for larger peptides of length of tens of amino acids (see Methods for more details).

We used the following reaction of zinc ion complexation (highlighting the two – potentially metal‐binding – protic thiol groups in **P1**):
(1)

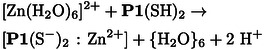




According to this equation, we computed the corresponding free energy change, Δ*G*_complexation_, as a sum of free energies of products minus a sum of free energies of the reactants. This complexation energy is related to the dissociation constant via Δ*G*_complexation_=−*RT*ln*K*_D_. The [Zn(H_2_O)_6_]^2+^ corresponds to the correct hydration number and coordination geometry of the metal ion.[^60^, ^61^, ^62^, ^63^] In the [**P1**(S^−^)_2_ : Zn^2+^] complex, the zinc ion is bound in a tetrahedral geometry without any water ligands, as these are forced out due to steric reasons. The presumable global minimum of [**P1**(S^−^)_2_ : Zn^2+^] was obtained by re‐optimizing 10 different structures from the MD at the DFT level. The same procedure was applied for the free **P1**(SH)_2_, which we obtained by DFT re‐optimization of 100 MD structures. For the water cluster {H_2_O}_6_, we used the energetically lowest conformation of a cluster of six waters, which we took from The Cambridge Cluster Database^[64]^ and optimized them on the same level. For the solvation free energy of H^+^, we used the value of −265.9 kcal mol^−1^.[^65^, ^66^] The final computed Δ*G*_complexation_ value at the B3LYP−D3/dgauss‐dzvp//COSMO‐RS level for reaction (1) is −22.4 kcal mol^−1^, more negative than the experimental (ITC) value of −9.1 kcal mol^−1^. Concerning the complexity of the system, this is a qualitatively correct result. The potential source of discrepancy is discussed in more detail below.

### 2.8 Structure Prediction Employing AlphaFold 3 and PEP‐FOLD4

To gain insight into the different behaviors of peptides **P1** and **P1C**, we have also done several structural predictions using AlphaFold 3 (AF3)^[29]^ and PEP‐FOLD4.^[67]^ Both peptides might be quite flexible in solution, so the predictions should be taken with a grain of salt.

We have used AF3 to predict the structure of peptides **P1** and **P1C** without zinc ion (Figure S12 in the SI). In all predictions, AF3 predicts a disulfide bond between the two cysteine residues. This is not surprising, as many natural short peptides, such as toxins, are held together by disulfide bridges. In the free forms, the predictions for **P1C** are more variable than those for **P1**, suggesting lower pre‐organization of the binding site of the control peptide. In general, for **P1C**, AF3 suggests a variable amount of α‐helix present between residues N2‐D13. However, MD shows a helical region in the latter half of the peptide (S11‐H19). In contrast, predictions for **P1** are more similar to one another and show α‐helices for residues M2‐R11 and D13‐H20, the latter of which aligns with the original design and the results from NMR.

In the AF3 predictions of the bound forms, all four presumed binding residues indeed bind the zinc ion in both [**P1** : Zn^2+^] and [**P1C** : Zn^2+^] (Figure S13 in the SI). H20 is also predicted to bind zinc ion in most models. Interestingly, the predictions for [**P1** : Zn^2+^] are more varied in terms of secondary structure content than for [**P1C** : Zn^2+^]. All of the models predict helical conformation for residues D13‐H18, which is consistent with the original design and NMR results. The models also predict various amount of α‐helix for residues M2‐R11, similarly as in the unbound predictions. For the complex [**P1C** : Zn^2+^], all predictions are roughly the same. They predict two helical regions, M3‐F7 and E12‐A21. The latter is in line with the final structure of the MD run, in which an α‐helix is observed for residues S11‐H19. As can be seen from Figure S13 in the SI, for both [**P1** : Zn^2+^] and [**P1C** : Zn^2+^], the conformation of the binding site is conformationally quite variable.

We have also decided to use a force‐field based oligopeptide structure predictor, PEP‐FOLD4. The default settings were employed, except that both termini were blocked (capped) and the Debye‐Hückel contribution was used. The best five predictions of peptide **P1** show mostly α‐helical content (Figure S14A in the SI). They all show S12‐C22 as helical, which is in line with original design and NMR structures, as well as AF3 predictions. Some models also predict α‐helix for parts of the region of M2‐F9, similarly to AF3, and one prediction predicts the whole peptide as a long α‐helix. For the peptide **P1C**, the best five models are essentially the same and they all predict α‐helix in the regions N2‐F7 and S11‐A22 (Figure S14B in the SI). PEP‐FOLD4, however, predicts higher secondary structure content than we observed in both ECD and NMR for both peptides.

## 3 Discussion

We have designed novel zinc‐binding peptides by merging fragments of proteins with known structures (deposited in the PDB). To simplify the problem and avoid the combinatorial complexity of designing a general {**B**_*k,l,m,n*_} metal‐binding site (where **B**_*i*_ is any of approximately 10 metal‐binding ‘natural’ AA side chains), we decided to limit ourselves to {C_2_H_2_} sites, i. e., zinc‐finger binding motifs. This allowed us to focus more on the design strategy and evaluation of binding properties of the ensuing peptides rather than selection of ‘ideal’ {**B**_*k,l,m,n*_} coordination site. We thus searched for potential H/C‐X_*i*_‐H/C metal‐binding fragments with suitable geometrical properties, including length of sequence and distance between presumable binding residues. The search was not restricted only to metalloproteins, the fragment candidates could have originated from *any* protein in PDB. After creating the fragment library, we attempted to merge various combinations of three fragments spanning the four metal‐ion coordination sites into a longer polypeptide. Employing various rational and computational criteria, we have selected three designed peptides for experimental validation of our approach. As a control experiment, we have also conceived three other peptides with similar sequences to validate the design process.

For **P1**, ITC has indeed confirmed that it is a very good zinc‐binder, with *K_D_* of approximately 220 nM. ITC also confirmed that about two protons are released upon Zn^2+^ binding, suggesting that **P1** binds the ion via two cysteine residues. Interestingly, binding of zinc to **P1** was favored both enthalpically and entropically. For the control peptide **P1C**, which consisted of the same fragments but with scrambled sequences, zinc‐binding was still intact, but the dissociation constant *K_D_* was about 2.5‐times higher compared to **P1** (520 nM compared to 220 nM). This means that the designed sequence was indeed a better binder than the control sequence. Both peptides bound zinc in a 1 : 1 stoichiometry, which agrees with the design of **P1**.

The structure of the [**P1** : Zn^2+^] complex was further probed by using NMR and ECD. Comparing NMR spectra of **P1** and [**P1** : Zn^2+^], we observed a clear difference between the two. The spectra clearly show that zinc binding affects the average conformation of the peptide chains, but according to ECD, the secondary structure content does not change much. Lack of any NMR cross peaks between the binding residues, however, suggests flexibility of the binding region. This agrees with ECD spectra, which showed that the free peptide is highly disordered. For **P1C**, we were not able to assess the structure using NMR, because the 1D spectra showed more peaks than would be expected, suggesting aggregation of the peptide. Upon addition of zinc ions, the disappearance of some peaks suggests either rearrangement of the aggregates, or their partial unfolding and dissolution.

The MD study of the structural behavior of **P1** in the bound form also agrees with the results of NMR. In the unbound form, however, the peptide is unstable and seems to refold into a different meta‐stable fold instead of a random coil. This all shows that indeed, the binding of zinc stabilizes the overall fold, similar to the case of zinc fingers. Similar conclusions can be made for **P1C**, which is stable in the [**P1C** : Zn^2+^] complex according to the MD simulation, but refolds into a different, more compact structure without the Zn^2+^ ion present. This supports the NMR results, where aggregation is observed for the unbound peptide. Upon addition of zinc ions in NMR, disappearance of peaks suggests (partial) unfolding of this initial structure. This observation would also be consistent with ITC measurements, in which **P1C** has a higher dissociation constant – more stable structure without zinc ions would require higher zinc concentration to accommodate the zinc ions.

We further assessed the dissociation constants of **P1** from a theoretical perspective using DFT calculations. The computed Δ*G*_complexation_ according to the simplified DFT‐D3/COSMO‐RS protocol is −22.4 kcal mol^−1^. While this is of the same order of magnitude as the experimental value from ITC (−9.1 kcal mol^−1^), the difference is rather large. The error may originate from the expectedly greater conformational entropy of the free peptide compared to its metal‐bound form, resulting in a value of the computed Δ*G*_complexation_ that is too negative. Furthermore, we were unable to perform *in vacuo* optimization, which is required for the complete protocol used in our previous studies. Finally, we admit that our structure of *apo* **P1** might not be a true global minimum, given the vastness of its conformational space. This result, however, is important from a computational point of view, as it illustrates that quantitatively accurate first principles calculations of complexation energies of larger oligopeptides are still not within the full grasp of computational chemistry. Nevertheless, the calculation correctly predicts that the formation of the [**P1** : Zn^2+^] complex is thermodynamically favored and spontaneous, which is in line with the experimental results.

Structure predictions made with AlphaFold 3 and PEP‐FOLD4 agree with both the designs and the observed structures (from CD, NMR, and MD) in terms of secondary structure content. Interestingly, AlphaFold 3 predicts **P1C** as more variable, whereas PEP‐FOLD4 predicts **P1** as more variable. This discrepancy, however, might originate from the presence of disulfide bridges in the AlphaFold 3 predictions. In the bound forms, as predicted by AlphaFold 3, the [**P1C** : Zn^2+^] complex is more rigid, which suggests it might not be able to accommodate the zinc ion as well as the [**P1** : Zn^2+^] complex. This could hint at a possible reason for the higher *K_D_* of **P1C**.

Peptides **P2** and **P3** were insoluble or bound zinc in a stoichiometry higher than 1 : 1 (more than one zinc ion bound to one peptide), respectively. We have thus not measured their binding constants with ITC. Their behavior might be influenced by additional cysteine residues present in their sequence, which might bind zinc ion in unanticipated ways. Peptide **P2C** seemed to aggregate and formed complexes with zinc in stoichiometry 1 : 2 (two peptides bound one zinc ion). Peptide **P3C** was also able to bind zinc, albeit with a higher *K_D_* (4.3 μM). Interestingly, this binding was enthalpically unfavorable and was compensated entropically. Therefore, we did not attempt to confirm the structure of peptides **P2**, **P3**, **P2C**, and **P3C**.

Out of the three selected peptides studied in this work, only one proved to be a successful design. This is quite likely because of presence of multiple cysteine residues in the sequences of the other two peptides. To improve this design methodology, we could limit sequences to only those without cysteine, as well as modify the selection process of individual designs before the experimental verification.

## 4 Conclusions

We have designed zinc(II)‐binding peptides similar to zinc fingers by taking fragments of various proteins and piecing them together. Several designs were tested experimentally, the best of which is **P1** with a 220 nM dissociation constant with Zn^2+^ ions. The favorable zinc binding is also correctly predicted by the DFT calculated complexation energy. This experimental proof shows the viability of this design process. For the next step, we want to explore the potential of this design process to design small peptides binding various metal ions as well as to design catalytic metallopeptides for various applications. At the same time, we want to extend our concept into designing more general metal‐binding sites, not being restricted to tetrahedral geometry, zinc(II) ion, and {C_2_H_2_} binding motif.

## Materials and Methods

### Peptide Synthesis

All peptides were assembled in a solid‐phase Liberty Blue synthesizer (CEM, Charlotte, NC, USA) by stepwise coupling of the corresponding Fmoc‐amino acids to the growing chain on Rink Amide MBHA resin (substitution 0.67 mmol/g). OXYMA/DIC in DMF was used as a coupling reagent. The fully protected peptide was synthesized, peptide was cleaved from the resin by mixture TFA/anisole/thioanisole/ethanedithiol (90 : 2 : 5 : 3). The TFA filtrate was evaporated at room temperature by nitrogen. The residue was precipitated with *tert*‐butyl methyl ether, collected by suction, dissolved in acetonitrile/water and lyophilized.

The crude peptide was purified by preparative HPLC using a C18 column (5 μm, 20×250 mm, Dr. Maisch). The purity and identity of the peptide were determined by analytical HPLC (column Symmetry, 3.5 μm, 4.6×75 mm, WATERS) and using ultrafleXtreme MALDI‐TOF/TOF (Bruker Daltonics, Germany).

### NMR Measurements

Samples of the peptides **P1** and **P1C** (1 mM, 160 μL) were dissolved in deuterated Tris buffer with pH 7.5 (10 % D_2_O, 90 % H_2_O) and measured either free or upon addition of 5 mM ZnCl_2_. NMR spectra were acquired at 25 °C using the 850 MHz Bruker Avance III HD instrument equipped with a triple‐resonance (15 N/13 C/1H) cryoprobe. Two dimensional heteronuclear (^15^N‐^1^H HSQC and ^13^C‐^1^H HSQC) and homononuclear (TOCSY – 55 ms mixing time and NOESY – 200 ms or 400 ms mixing time) spectra were measured, all pulse programs were from standard Bruker library. Spectra were processed in Bruker Topspin I and analyzed in NMRFAM‐SPARKY.^[68]^

^1^H−^1^H distance restraints were derived from the 2D NOESY spectrum using 400 ms mixing time. Structural calculation was carried out in CYANA^[69]^ using NOESY data in combination with backbone torsion angle restraints, generated from assigned chemical shifts using the program TALOS+.^[70]^ The combined automated NOE assignment and structure determination protocol (CANDID) was used for automatic NOE cross‐peak assignment. Subsequently, five cycles of simulated annealing combined with redundant dihedral angle restraints were used to calculate a set of converged structures with no significant restraint violations (distance and van der Waals violations <0.5 Å and dihedral angle constraint violations<5°).

### Electronic Circular Dichroism

Electronic circular dichroism (ECD) spectra were measured on a Jasco 815 spectropolarimeter over a spectral range of 190 nm to 280 nm. Sample concentration was kept the same as for NMR experiments, i. e., 1 mM for both peptides **P1** and **P1C**. Peptides were dissolved in Tris buffer at pH 7.5 and measured either free or upon addition of 5 mM ZnCl_2_ (for [peptide:Zn^2+^] complex in stoichiometry 1 : 1) or 10 mM ZnCl_2_ (for [peptide : Zn^2+^] complex in stoichiometry 1 : 2). For the measurements in far‐UV spectral region, a cylindrical quartz cell with a path length of 0.02 cm and in near‐UV spectral region quartz cell with a path length of 0.2 cm were used. For both spectral regions, the measurements were done in the following setup: a scanning speed of 5 nm/min, a response time of 16 seconds, standard instrument sensitivity and 2 spectra accumulations. After a baseline correction, spectra were expressed in terms of molar ellipticity per residue (θ). The numerical analysis of secondary structures was performed using the CDPro software package.[^71^, ^72^, ^73^]

### Isothermal Titration Calorimetry

The bindings of ZnCl_2_ to peptides **P1**, **P1C**, and **P3C** were monitored at 25 °C using a VP‐ITC microcalorimeter (MicroCal Inc./Malvern Instruments Ltd., UK). Reactant solutions were prepared in 20 mM buffer at pH 7.0 and the exact concentrations of peptides were determined by HPLC amino acid analysis. Typically, 9 μl aliquots of 650 μM ZnCl_2_ were injected stepwise into a sample cell containing 1.43 mL of 50 μM peptide until saturation was achieved. To estimate whether binding of Zn^2+^ to peptide was accompanied by proton transfer, titrations in four buffers with different ionization enthalpies (sodium cacodylate, PIPES, HEPES and ACES) were performed.^[56]^ Experimental titrations were accompanied by a corresponding control experiment, where solely the ligand was injected into the buffer. The thermodynamic parameters were determined by MicroCal software implemented in Origin 7.0 (MicroCal Inc./Malvern Instruments Ltd., UK).

The observed enthalpy change (Δ*H*_obs_) can be expressed as a combination of the binding enthalpy (Δ*H*_bind_) and the enthalpy of ionization of the buffer (Δ*H*_ion_) using the relationship:
(2)

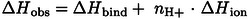




By performing the experiment at a constant pH using buffers with different ionization enthalpies (Δ*H*_ion_), the binding enthalpy (Δ*H*_bind_) can be determined. By plotting experimental enthalpies against the ionization enthalpies of the used buffers (Figure 3B and C), the slope of the straight line corresponds to the negative of the (average) number of protons released during the formation of the peptide–ion complex (*n*_H+_). The interception of the straight line with the *y*‐axis then determines the buffer corrected binding enthalpy. If the heat of buffer protonation is not considered when determining the reaction enthalpy, the resulting evaluation of entropic and enthalpic contributions to Gibbs energy may yield to inaccurate or misleading values.[^57^, ^74^]

### Molecular Dynamics Simulations

For the processing of the 101 designed peptides, we prepared MD simulations using psfgen module of VMD^[75]^ in two versions: (i) with zinc ion ligated by two deprotonated cysteine residues and two histidine residues, and (ii) metal‐free version where the cysteines are present in the standard neutral form. For these MD runs, standard parameters of CHARMM 36^[76]^ forcefield were used for the protein residues and the zinc ion. All other polar AAs were set to the standard protonation state at pH 7. The protein was solvated in a cubic water box (10 Å around the protein) with TIP3P water. Periodic boundary conditions were used to ensure continuity of the solvent environment. Charge of the peptides was not balanced with any counterions. NAMD^[77]^ was used to conduct the MD simulation. The MD protocol consisted of the following steps: (1) initial minimization (1000 steps), (2) heating from 0–298 K, (3) 100 ps of equilibration at the final temperature. 1 fs steps were used. The length of the simulation run was 4 ns.

The selected structures of zinc‐binding peptides **P1** and **P1C** were prepared using psfgen module of VMD as described above. Standard parameters of CHARMM 36 forcefield were also used. All other polar AAs were set to the standard protonation state at pH 7. The protein was solvated in a cubic water box (10 Å around the protein) with TIP3P water, and the solution was neutralized by a chloride anion. NAMD was also used to conduct the MD simulation protocol. Periodic boundary conditions were used to ensure continuity of the solvent environment. The MD protocol consisted of the following steps: (1) initial minimization (1000 steps), (2) heating from 0–298 K, (3) 100 ps of equilibration at the final temperature. 1 fs steps were used for equilibration, while 2 fs integration steps were used for the subsequent production simulations. For the subsequent QM calculations, we took 100 snapshots from 50–450 ns of the production simulation. Videos from the MD runs were visualized using VMD.

### Quantum Chemical Calculations

Initial structures for DFT optimizations were taken from the MD (see above): 100 snapshots of the free **P1** and 10 snapshots of the [**P1** : Zn^2+^] complex. These structures were optimized using BP86 functional[^78^, ^79^] with D3 dispersion[^80^, ^81^] with special parameters for proteins (file ‘disp.par’ containing ‘*1.0 0.7182 3.2176 3.8572 1.0 4*’, which correspond to parameters s6, rs6, s18, rs18, alpha, and version)^[82]^ and dgauss‐dzvp basis set (def2‐TZVP for zinc atom) employing COSMO solvation model[^83^, ^84^] as implicit solvent with *ϵ*_r_=80 (water). After the optimization, the lowest‐energy conformer, one for *apo* peptide and one for bound peptide, was selected as the global minimum for subsequent calculations. We then calculated the single point in *ϵ*_r_=∞ using FINE cavity with B3LYP functional[^79^, ^85^, ^86^] and dgauss‐dzvp basis set (for all atoms) with the same special D3 dispersion correction, resulting in a cavity file. For the same structure, we also calculated the electronic energy in gas‐phase (without solvent), *E_gas_*, on the same level. The results of these two single point DFT calculations (cavity file and gas‐phase energy) were then processed by COSMO‐RS[^87^, ^88^] using COSMOtherm2023 program, from which we obtained the solvation energy, *G_solv_*. We also used the DFT optimized geometry as the input for frequency calculations, which we calculated using xTB. First, the structure was reoptimized using GFN2‐xTB^[89]^ with water as implicit solvent using the analytical linearized Poisson‐Boltzmann (ALPB) model,^[90]^ then we calculated single point Hessian as implemented in xTB, which we used to calculate the thermal contribution, *E_ZPVE_–RTln(q_trans_q_rot_q_vib_)+pV*. All negative frequencies had wavenumbers less negative than −100 cm^−1^; these were treated as numerical inaccuracies and converted into positive values. The structures reoptimized with xTB did not differ significantly from the DFT optimized structures. The total free energy of the molecule was then taken as:
(3)

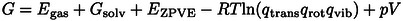




All DFT calculations were performed in Turbomole 7.7.^[91]^

### Other

All images were generated using PyMOL.^[92]^

## Author Contributions

Ján Michael Kormaník has overseen the manuscript preparation and contributed to the quantum chemical calculations. Daniel Herman has done the peptide design and pre‐screening for the best candidates for synthesis. Erik Andris has helped with analysis of the NMR and quantum chemical calculations. Martin Culka has done the MD study on the designed peptides. Ondrej Gutten has helped with analysis of the NMR and quantum chemical calculations. Milan Kožíšek has measured and analyzed the ITC data. Lucie Bednárová has measured and analyzed the ECD spectra. Pavel Srb has measured and analyzed the NMR spectra. Václav Veverka assisted in designing the NMR experiments. Lubomír Rulíšek has conceptualized the project, oversaw its realization, took lead in writing the manuscript, as well as contributed to the quantum chemical calculations.

## Conflict of Interests

The authors declare no conflict of interest.

5

## Supporting information

As a service to our authors and readers, this journal provides supporting information supplied by the authors. Such materials are peer reviewed and may be re‐organized for online delivery, but are not copy‐edited or typeset. Technical support issues arising from supporting information (other than missing files) should be addressed to the authors.

Supporting Information

## Data Availability

The data that support the findings of this study are available in the supplementary material of this article.
