# Supplementary material for: Design of Zn‐Binding Peptide(s) from Protein Fragments
Source: Chembiochem. 2025 Feb 26;26(7):e202401014. doi: 10.1002/cbic.202401014 (PMC12002108; doi:10.1002/cbic.202401014)
Supplement: Supplementary file 1 — Supporting Information [file CBIC-26-e202401014-s001.pdf]

ChemBioChem

Supporting Information

Design of Zn-Binding Peptide(s) from Protein Fragments

Ján Michael Kormaník, Daniel Herman, Erik Andris, Martin Culka, Ondrej Gutten, Milan Kožíšek, Lucie Bednárová, Pavel Srb, Václav Veverka, and Lubomír Rulíšek*

Design of Zn-binding Peptide(s) from Protein Fragments

Supporting Information

*Ján Michael Kormaník[†], Daniel Herman[†], Erik Andris, Martin Culka, Ondrej Gutten, Milan Kožíšek, Lucie Bednárová, Pavel Srb, Václav Veverka, Lubomír Rulíšek**

*[†] - equal contribution, * - corresponding author*

Institute of Organic Chemistry and Biochemistry of the Czech Academy of Sciences,

Flemingovo náměstí 2, 166 10, Prague 6, Czech Republic

Supporting Information. This material is available free of charge via the Internet at xxx.

1. Isothermal Calorimetry of Peptides P1, P1C, and P3C

Binding of Zn^{2+} ion to **P3C**, associated with the K_D = 4.3 μM, exhibits unfavorable binding enthalpy change (4.5 kcal·mol^{-1}) which was compensated by more favorable entropic contribution of -11.9 kcal·mol^{-1} (n_{H+} = 1.6; **Table S2**). This suggests somewhat different behavior in the interaction of **P3C** with Zn^{2+} ion compared to peptides **P1** and **P1C**.

The titration curves of all three peptides are shown in **Figures S1-S3**.

Table S1. Thermodynamic parameters of Zn^{2+} ion binding to peptide **P3C**.

Peptide	ΔG_{bind} [kcal·mol^{-1}]	ΔH_{bind} [kcal·mol^{-1}]	$-T\cdot\Delta S_{bind}$ [kcal·mol^{-1}]	K_A [M^{-1}]	K_D [nM]	stoichiometry	n_{H+}
**P3C**	-7.3 ± 0.3	4.5 ± 0.6	-11.8 ± 0.9	(2.3 ± 1.1) · 10^5	4 300 ± 2 500	1.1 ± 0.1	-1.6 ± 0.1

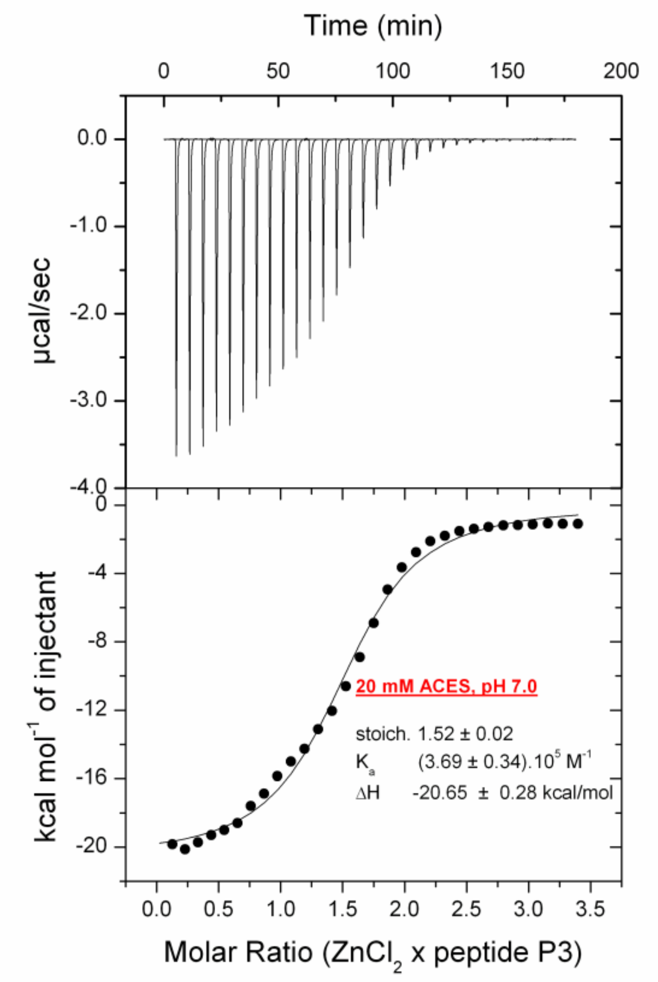

Figure S1. Isothermal titrations of **P3** with $ZnCl_2$ performed in 20 mM ACES, pH 7.0, showing stoichiometry 1.5.

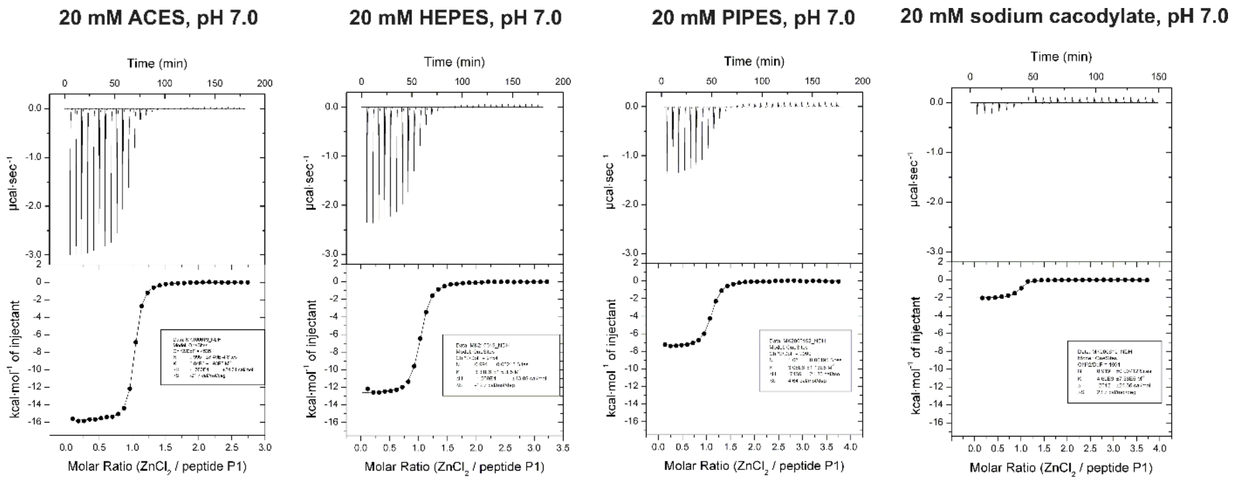

Figure S2. Isothermal titrations of **P1** with $ZnCl_2$ performed in buffers with different enthalpies of ionization ΔH_{ion} (ACES, HEPES, PIPES, and sodium cacodylate).

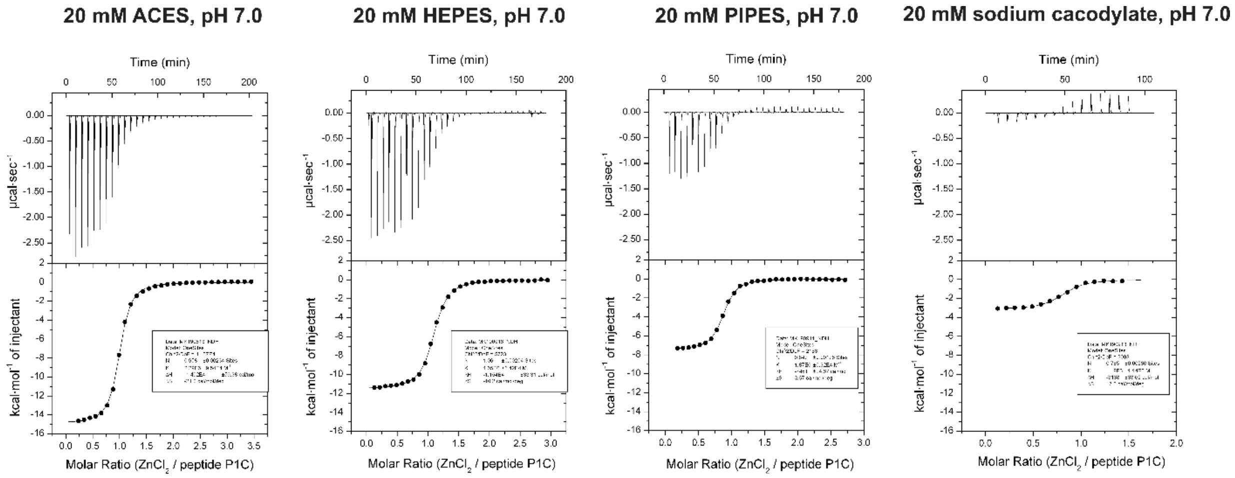

Figure S3. Isothermal titrations of **P1C** with $ZnCl_2$ performed in buffers with different enthalpies of ionization ΔH_{ion} (ACES, HEPES, PIPES, and sodium cacodylate).

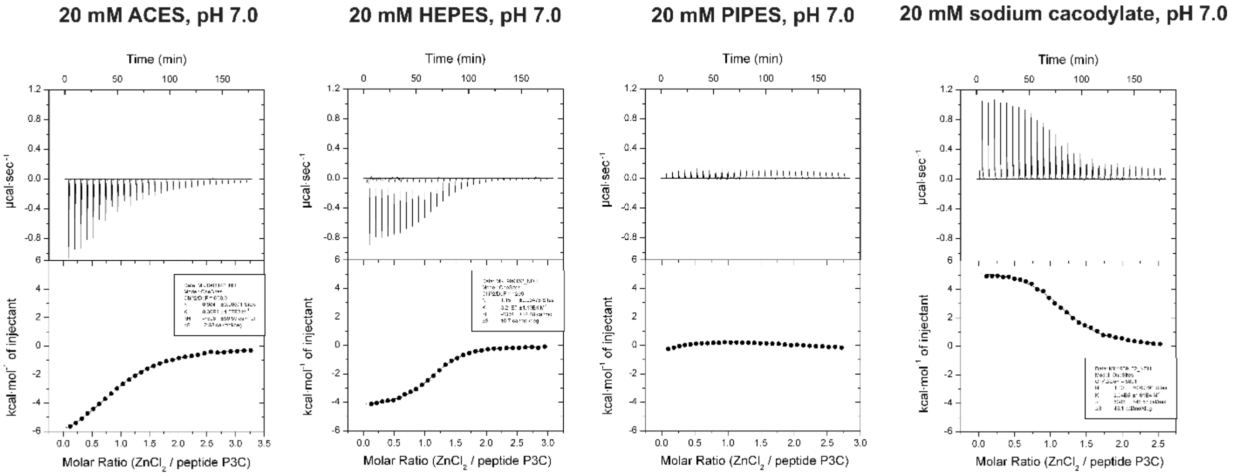

Figure S4. Isothermal titrations of **P3C** with $ZnCl_2$ performed in buffers with different enthalpies of ionization ΔH_{ion} (ACES, HEPES, PIPES, and sodium cacodylate).

2. Electronic Circular Dichroisms of Peptides P1 and P1C

Table S2. Numerical analysis of the ECD spectra of peptides **P1** and **P1C** using CDPro numerical fitting methods (see Refs 61-63 in the main text).

Peptide	α–helix (%)	β- structure (%)	β-turn (%)	disordered (%)
**P1**	8	37	22	33
**P1-Zn^{2+}(1:1)**	7	36	23	34
**P1-Zn^{2+}(1:2)**	7	38	23	32
**P1 (in 25% TFE)**	23	24	22	31

**P1C**	7	35	22	36
**P1C-Zn^{2+}(1:1)**	7	37	22	34
**P1C-Zn^{2+}(1:2)**	7	37	23	35
**P1C (in 25% TFE)**	23	25	17	25

3. NMR Data of Peptide P1

Table S3: NMR Constraints and Statistics for the final set of structures

**Non-redundant distance and angle constrains**	
Total number of NOE restraints	145
Intra-residue (i = j)	71
Sequential (| i – j | = 1)	66
Medium-range NOEs (1 < | i – j | < 5)	0
Long-range NOEs (| i – j | ≥ 5)	8
Torsion angles	16
Total number of restricting restraints	145
Total restricting restraints per restrained residue	6.6
**Residual constraint violations**	
Distance violations per structure	
0.1 – 0.2 Å	0.25
0.2 – 0.5 Å	0
> 0.5 Å	0
r.m.s. of distance violation per constraint	0.05 Å
Maximum distance violation	0.15 Å
Dihedral angle viol. per structure	
1 – 10 °	7.43
> 10 °	0
r.m.s. of dihedral violations per constraint	1.48°
**Ramachandran plot summary**	
Most favoured regions	52 %

Additionally allowed regions	47 %
Generously allowed regions	0.0 %
Disallowed regions	0.5 %
r.m.s.d. to the mean structure	All / ordered
All backbone atoms	2.1 / 0.4 Å
All heavy atoms	3.2 / 1.3 Å

Table S4. Long-range restraints used in NMR modeling. Interactions between F9 and E14/L15 constitute the hydrophobic core of the peptide. For protons, Q stands for equivalent (e.g. for F9, QD represents both HD1 and HD2). In the case of L15, QQD represents all 6 terminal protons of the side chain.

1st residue	Protons	2nd residue	Protons	Distance (Å)
F9	QD	E14	QB	5.34
F9	QD	E14	QG	5.50
F9	QE	E14	QB	5.12
F9	QE	E14	QG	5.01
F9	QE	L15	HA	5.50
F9	QE	L15	QQD	5.44
F9	HZ	E14	QB	5.34
F9	HZ	E14	QG	5.50

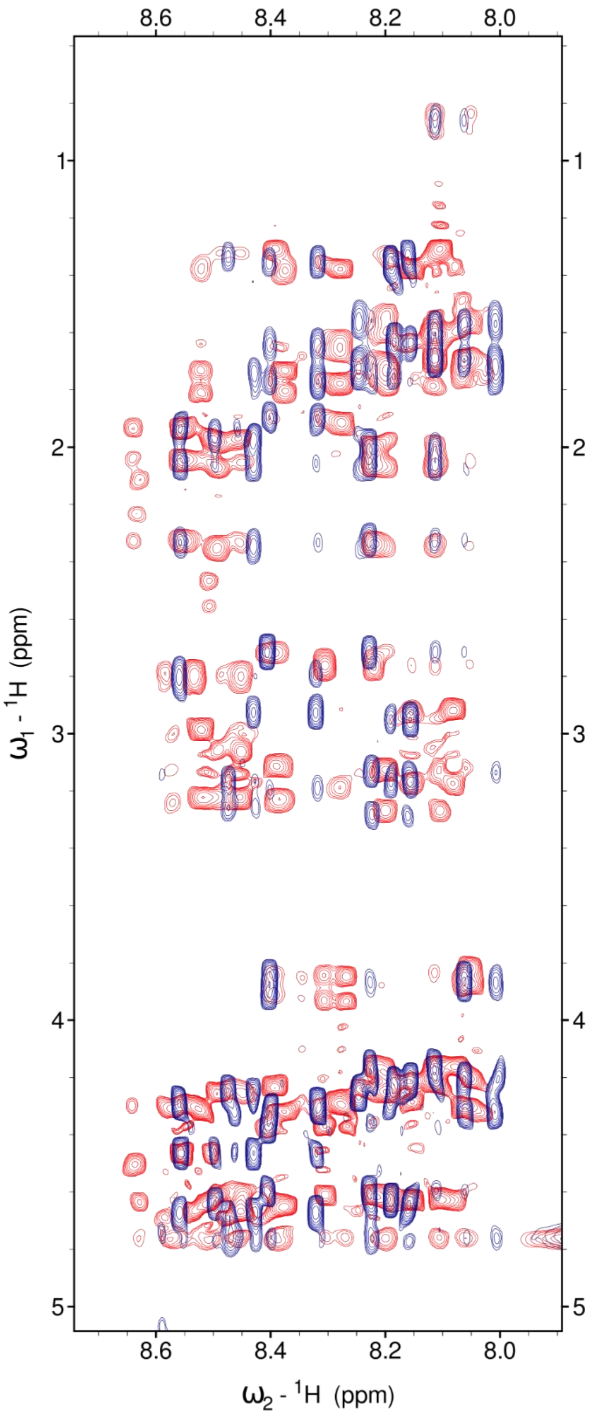

Figure S5. An overlay of 2D NOESY spectra of [**P1**:Zn^{2+}] complex (red) and **P1** without Zn^{2+} (blue).

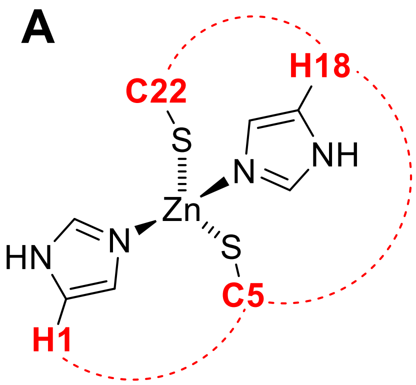

Figure S6. The geometrical isomers of the binding site. Due to the asymmetricity of the peptide, the binding residues can create two different binding sites around the zinc ion. According to NMR models, both can appear in solution and potentially interconvert between one another, which might explain why no contacts between the binding residues are observed. Peptide is shown schematically with N-terminus in bottom left and C-terminus in top left.

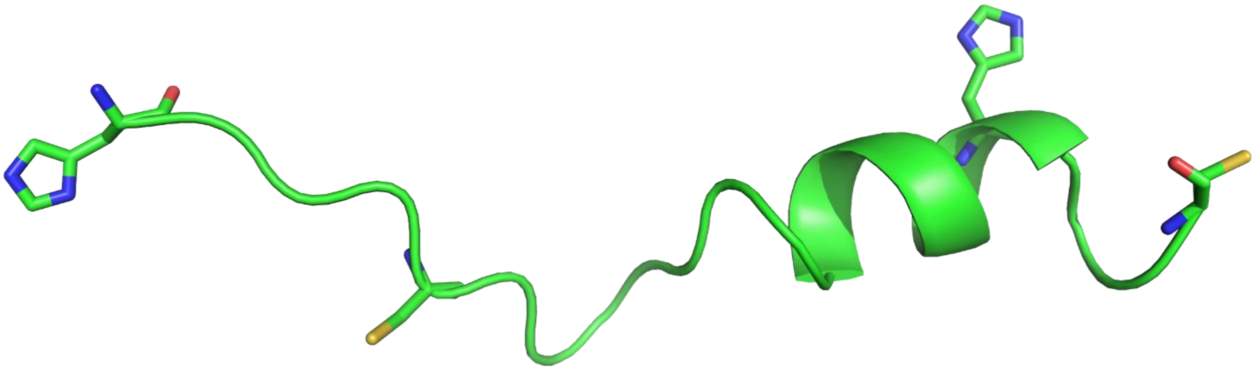

Figure S7. One of the NMR models of the unbound **P1**. Helical conformation is observed for residues D13-A19, the rest of the peptide is flexible. Binding residues are shown as sticks, hydrogens are omitted for clarity.

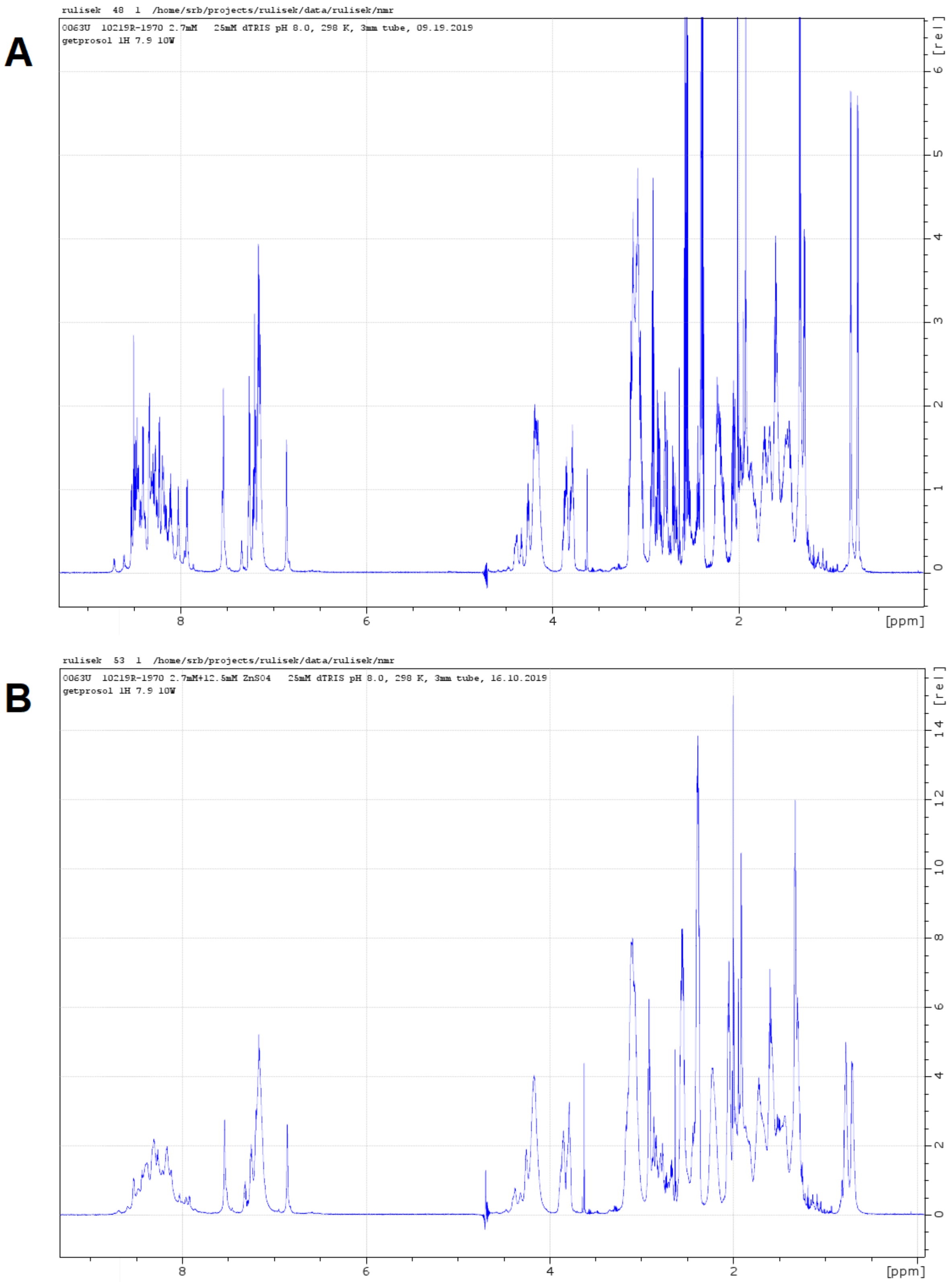

Figure S8. 1D NMR spectrum of (A) **P1C** and (B) [**P1C**:Zn^{2+}] in 10% D_2O, 90% H_2O.

4. Molecular dynamics data of peptide P1

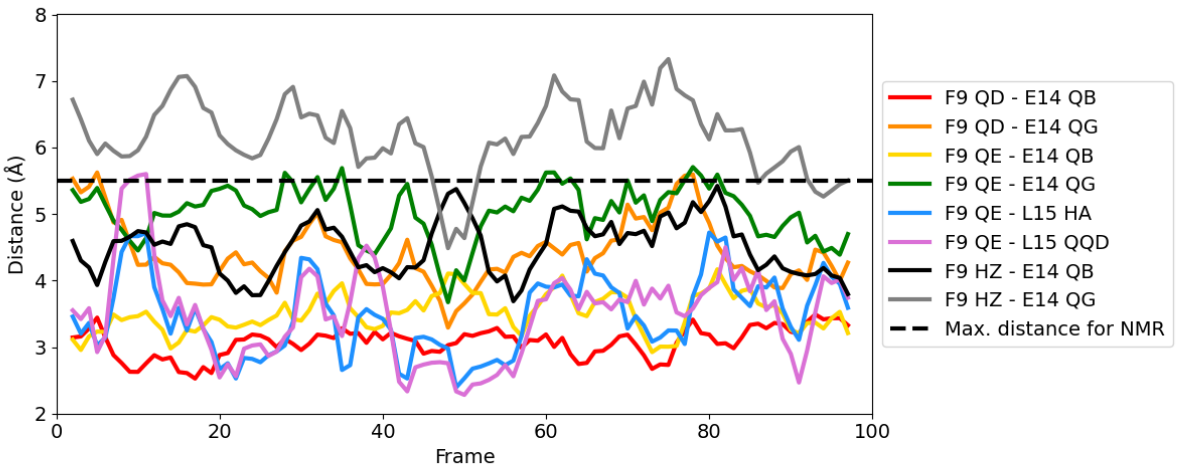

Figure S9. Plot of the distances between aromatic protons of F9 and side chain protons of E14/L15 during the MD run of **P1**. (For more details about specific pairs of protons, see Table S4 above.) For equivalent protons (e.g., F9 QD representing both HD1 and HD2), the shortest measured distance is shown. Most of the distances are, on average, shorter than 5.5 Å (dashed black line), confirming the fact that they are visible in NMR. Moving average over five frames is plotted.

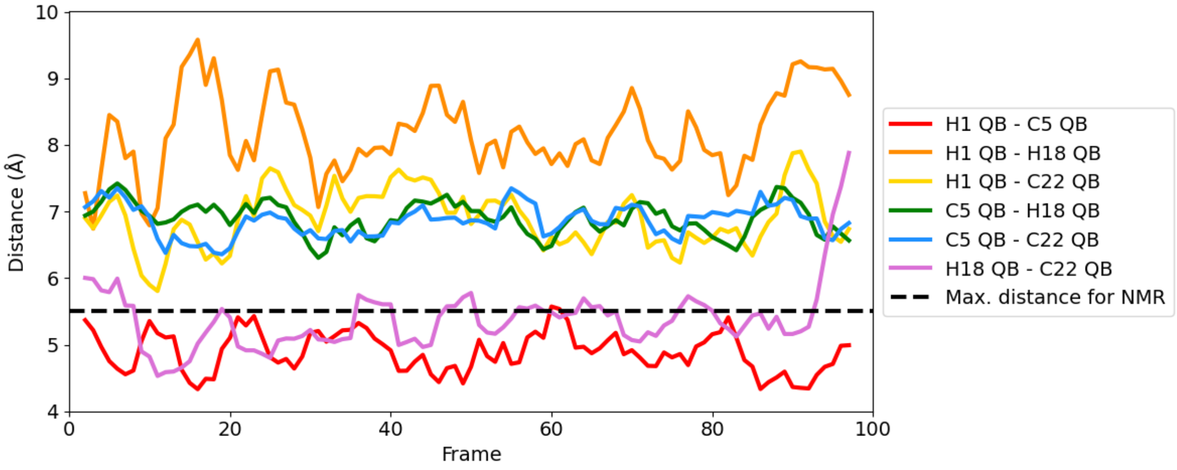

Figure S10. Plot of the distances between side chain β-protons of the binding residues (H1, C5, H18, C22) during the MD run of **P1**. For equivalent protons (e.g., H1 QB representing both HB1

and HB2), the shortest measured distance is shown. Most of the distances are on average longer than 5.5 Å (dashed black line), meaning they indeed should not be visible in NMR. Moving average over five frames is plotted.

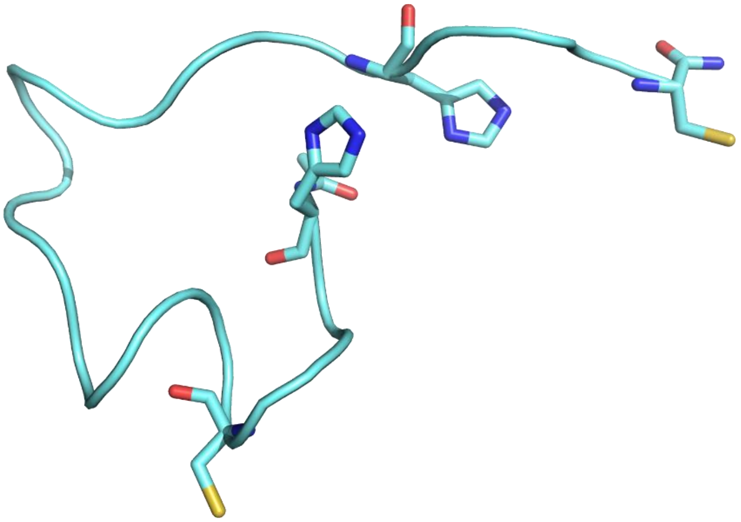

Figure S11. The meta-stable structure from MD study of **P1** without zinc. The peptide unfolds during the MD run and after about 250 ns (midway through the simulation), it refolds into the depicted alternate meta-stable structure, in which it remains until the end of the simulation. Interestingly, this structure contains a left-handed alpha-helix formed by residues E4-A11 (bottom). The peptide is shown by cartoon with binding residues shown as sticks, hydrogens are omitted for clarity.

5. AlphaFold 3 and PEP-FOLD4 structure predictions

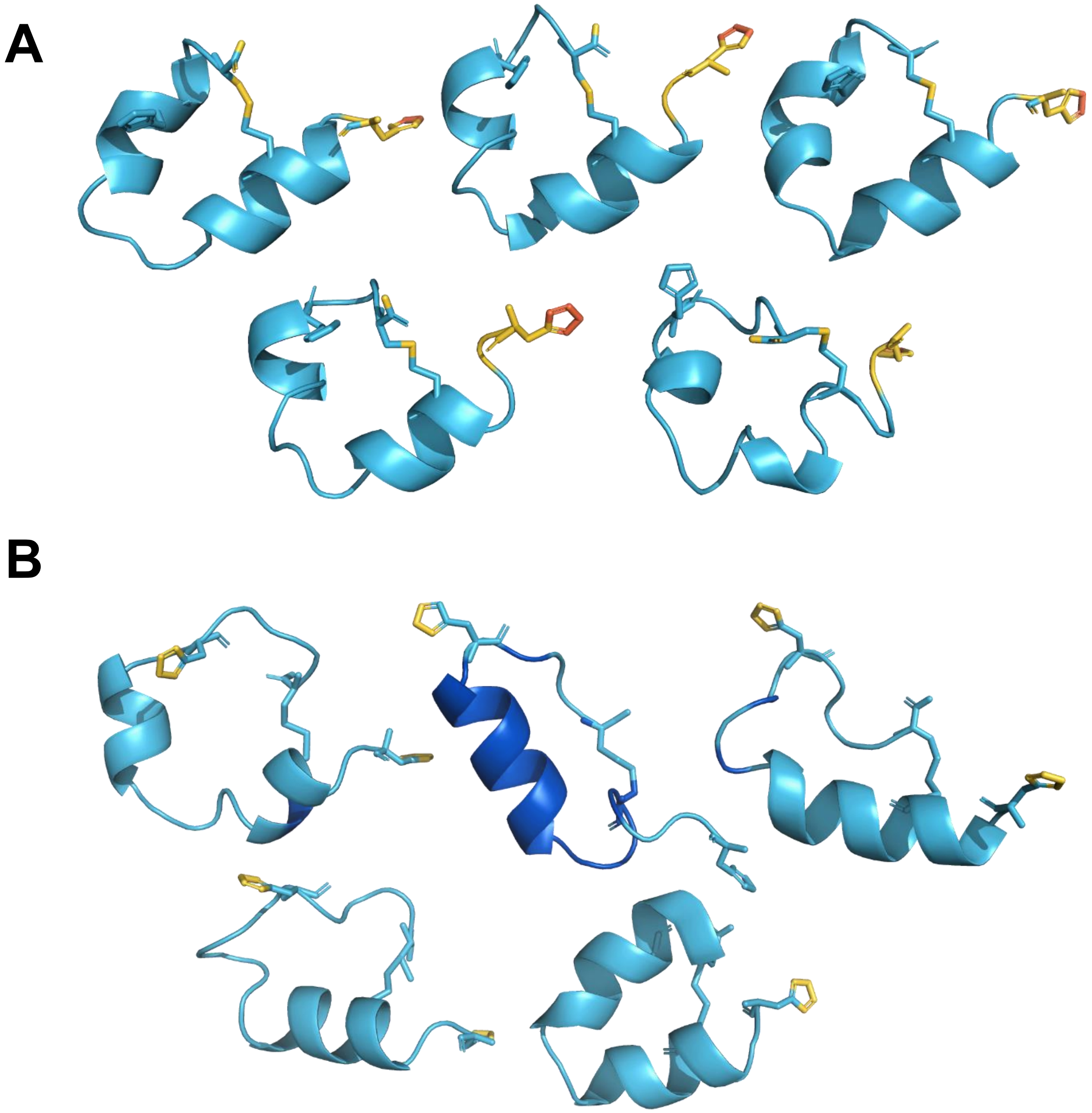

Figure S12. The best 5 models of structure of **P1** (**A**) and **P1C** (**B**) as predicted by AlphaFold 3. Structures are colored by AlphaFold pLDDT confidence and shown by cartoon with proposed binding residues in sticks, hydrogens are omitted for clarity.

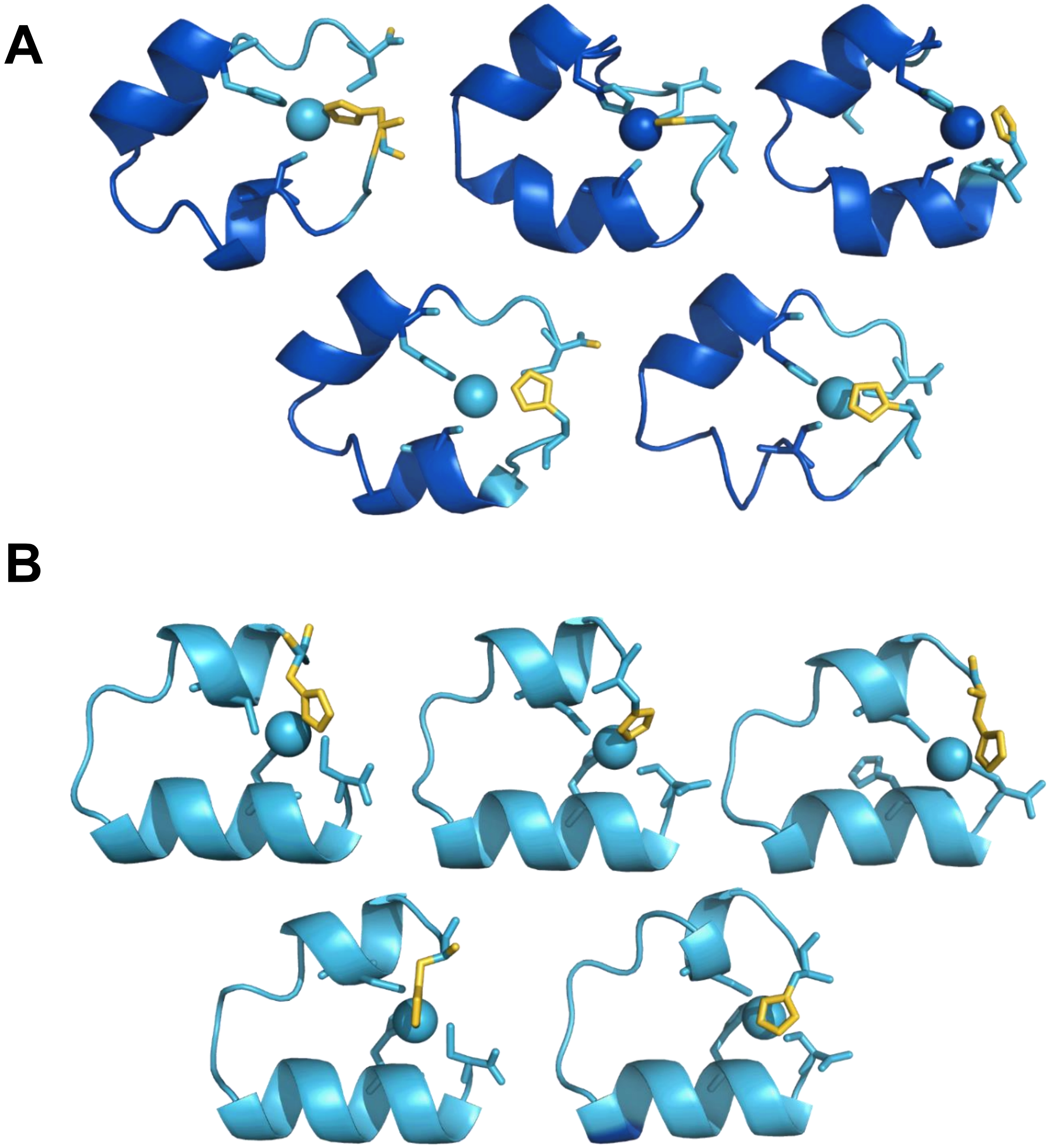

Figure S13. The best 5 models of structure of the complexes [**P1**:Zn^{2+}] (**A**) and [**P1C**:Zn^{2+}] (**B**) as predicted by AlphaFold 3. Structures are colored by AlphaFold pLDDT confidence and shown by cartoon with proposed binding residues in sticks, zinc ion is shown as a sphere, hydrogens are omitted for clarity.

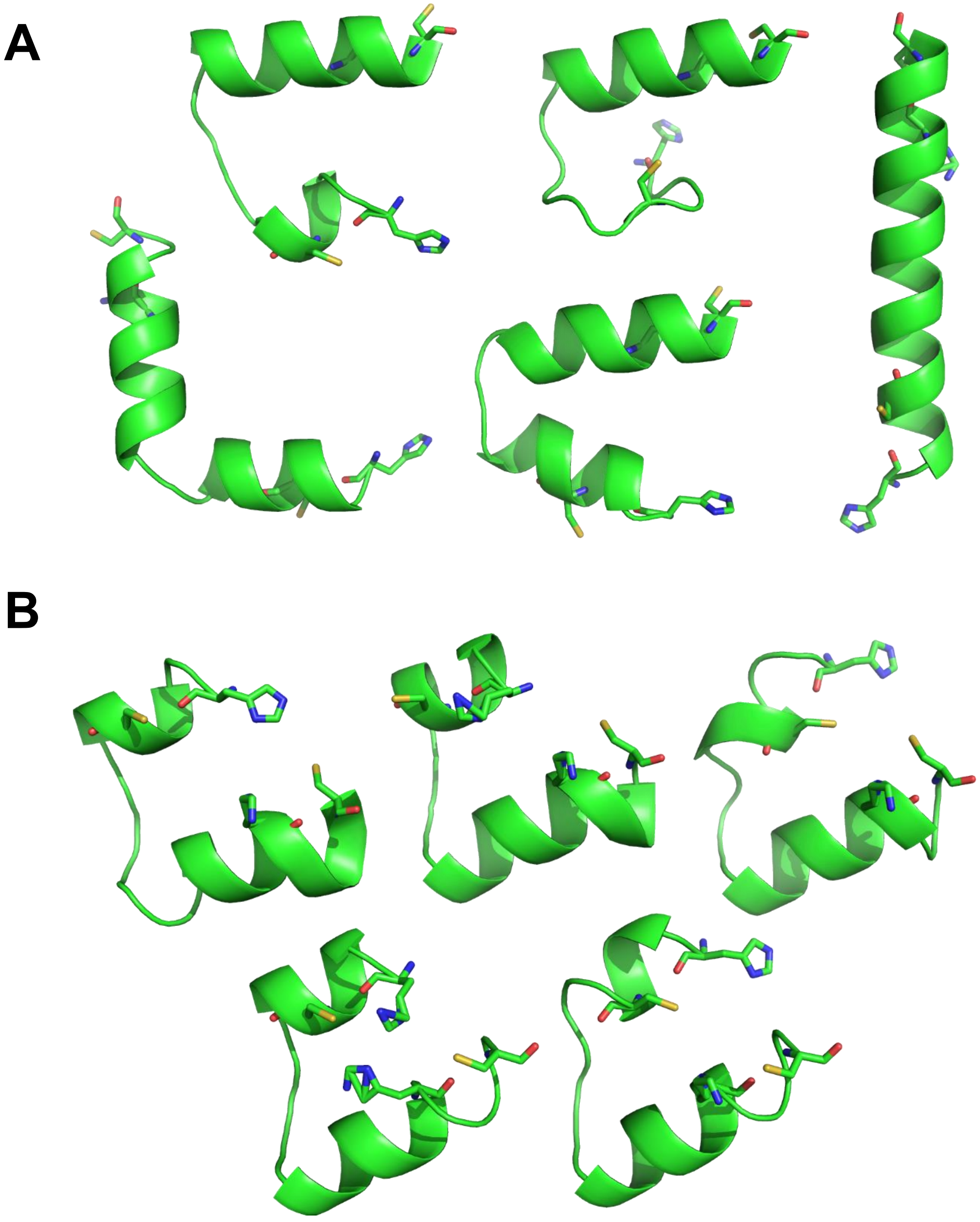

Figure S14. The best 5 models of structure of **P1** (**A**) and **P1C** (**B**) as predicted by PEP-FOLD4. Structures are shown by cartoon with proposed binding residues in sticks, hydrogens are omitted for clarity.
